# Supplementary material for: Mechanical ventilation for COVID-19: Outcomes following discharge from inpatient treatment
Source: PLoS One. 2023 Jan 6;18(1):e0277498. doi: 10.1371/journal.pone.0277498 (PMC9821470; doi:10.1371/journal.pone.0277498)
Supplement: S1 File — (DOCX) [file pone.0277498.s001.docx]

**Supplemental Tables**

**Supplemental Table 1:** Categories for Reasons for Readmission with Associated ICD-10 codes

|  | | ICD-10  Code Group 1 | ICD-10  Code Group 2 | ICD-10  Code Group 3 |
| --- | --- | --- | --- | --- |
| Reason for Readmission | Abnormal Symptoms and Labs | R00 to R99 |  |  |
|  | Birth | P00 to P96 | Q00 to Q99 |  |
|  | Blood Disease | C00 to C96 | D00 to D89 |  |
|  | Circulatory Issues | I00 to I99 |  |  |
|  | COVID-19 | U07.1 | J12.82 |  |
|  | Digestive | K00 to K95 |  |  |
|  | Endocrine | E00 to E89 |  |  |
|  | Eyes, Ears, and Skin | H00 to H59 | H60 to H95 | L00 to L99 |
|  | Genitourinary | N00 to N99 |  |  |
|  | Infectious Disease | A00 to A99 | B00 to B99 |  |
|  | Injury | S00 to S99 | T07 to T88 |  |
|  | Mental | F01 to F99 |  |  |
|  | Muscular | M00 to M99 |  |  |
|  | Nervous System | G00 to G99 |  |  |
|  | Other | U00 to U85 | V00 to Y99 | Z00 to Z99 |
|  | Pregnancy | O00 to O99 |  |  |
|  | Respiratory | J00 to J99 |  |  |

**Supplementary Table 2**: Absolute Standardized Differences Between Treated and Untreated Samples Prior to Propensity Score Matching/Inverse Probability Weighting

|  | | | Total (n=17,562) | Did Not Receive Mechanical Ventilation (n=16,431) | Received Mechanical Ventilation (n=1,131) | Absolute Standardized Difference (ASD) |
| --- | --- | --- | --- | --- | --- | --- |
| Month of Admission | March 2020 through April 2020 | | 8,674 (49.4%) | 7,902 (48.1%) | 772 (68.3%) | 0.42 |
|  | May 2020 through July 2020 | | 1,862 (10.6%) | 1,792 (10.9%) | 70 (6.2%) |  |
|  | August 2020 through October 2020 | | 888 (5.1%) | 849 (5.2%) | 39 (3.4%) |  |
|  | November 2020 through January 2021 | | 6,138 (35.0%) | 5,888 (35.8%) | 250 (22.1%) |  |
| **Demographics** | | | | | | |
| Age; Median (IQR) | | | 63 (25) | 63 (26) | 63 (19) | 0.02 |
| Sex | | Female | 8,280 (47.1%) | 7,878 (47.9%) | 402 (35.5%) | 0.25 |
|  |  | Male | 9,282 (52.9%) | 8,553 (52.1%) | 729 (64.5%) |  |
| Race | | White | 7,597 (43.3%) | 7,148 (43.5%) | 449 (39.7%) | 0.15 |
|  |  | Black | 3,231 (18.4%) | 3,056 (18.6%) | 175 (15.5%) |  |
|  |  | Asian | 1,479 (8.4%) | 1,367 (8.3%) | 112 (9.9%) |  |
|  |  | Other/Multiracial | 4,505 (25.7%) | 4,172 (25.4%) | 333 (29.4%) |  |
|  |  | Unknown/Missing | 750 (4.3%) | 688 (4.2%) | 62 (5.5%) |  |
| Ethnicity | | Hispanic/Latinx | 3,411 (19.4%) | 3,157 (19.2%) | 254 (22.5%) | 0.13 |
|  |  | Non-Hispanic | 13,164 (75.0%) | 12,373 (75.3%) | 791 (69.9%) |  |
|  |  | Other/Unknown | 987 (5.6%) | 901 (5.5%) | 86 (7.6%) |  |
| Insurance | | Commercial | 6,156 (35.1%) | 5,736 (34.9%) | 420 (37.1%) | 0.1 |
|  |  | Medicare | 7,185 (40.9%) | 6,769 (41.2%) | 416 (36.8%) |  |
|  |  | Medicaid | 3,809 (21.7%) | 3,537 (21.5%) | 272 (24.0%) |  |
|  |  | Self-Pay | 114 (0.6%) | 109 (0.7%) | 5 (0.4%) |  |
|  |  | Other | 298 (1.7%) | 280 (1.7%) | 18 (1.6%) |  |
| **Comorbidity** | | | | | | |
| Smoking Status | | Current | 435 (2.5%) | 417 (2.5%) | 18 (1.6%) | 0.4 |
|  |  | Former | 1,975 (11.2%) | 1,859 (11.3%) | 116 (10.3%) |  |
|  |  | Never | 12,990 (74.0%) | 12,291 (74.8%) | 699 (61.8%) |  |
|  |  | Unknown | 1,938 (11.0%) | 1,664 (10.1%) | 274 (24.2%) |  |
|  |  | Missing | 224 (1.3%) | 200 (1.2%) | 24 (2.1%) |  |
| Asthma | | | 1,235 (7.0%) | 1,144 (7.0%) | 91 (8.0%) | 0.04 |
| COPD | | | 1,022 (5.8%) | 958 (5.8%) | 64 (5.7%) | 0.01 |
| Obstructive Sleep Apnea | | | 563 (3.2%) | 509 (3.1%) | 54 (4.8%) | 0.09 |
| Hypertension | | | 8,682 (49.4%) | 8,066 (49.1%) | 616 (54.5%) | 0.11 |
| Myocardial Infarction | | | 280 (1.6%) | 239 (1.5%) | 41 (3.6%) | 0.14 |
| Heart Failure | | | 1,411 (8.0%) | 1,297 (7.9%) | 114 (10.1%) | 0.08 |
| Stroke / Ischemic Disease | | | 242 (1.4%) | 220 (1.3%) | 22 (1.9%) | 0.05 |
| Aortic Aneurysm | | | 57 (0.3%) | 55 (0.3%) | 2 (0.2%) | 0.03 |
| CVD (all) | | | 1,607 (9.2%) | 1,464 (8.9%) | 143 (12.6%) | 0.12 |
| Diabetes Mellitus | | | 792 (4.5%) | 730 (4.4%) | 62 (5.5%) | 0.05 |
| CKD | | | 1,826 (10.4%) | 1,701 (10.4%) | 125 (11.1%) | 0.02 |
| Cancer | | | 1,442 (8.2%) | 1,359 (8.3%) | 83 (7.3%) | 0.03 |
| Dementia | | | 1,224 (7.0%) | 1,186 (7.2%) | 38 (3.4%) | 0.17 |
| Immunodeficiency | | | 88 (0.5%) | 80 (0.5%) | 8 (0.7%) | 0.03 |
| **Visit Details** | | | | | | |
| Length of Stay; Median (IQR) | | | 5 (7) | 5 (6) | 19 (27) | 1.05 |
| Antiviral Treatment | | | 9,955 (56.7%) | 9,032 (55.0%) | 923 (81.6%) | 0.6 |
| Anticoagulant Treatment | | | 15,887 (90.5%) | 14,766 (89.9%) | 1,121 (99.1%) | 0.41 |
| Corticosteroid Treatment | | | 8,651 (49.3%) | 7,760 (47.2%) | 891 (78.8%) | 0.69 |
| IL-1 Inhibitor Treatment | | | 673 (3.8%) | 520 (3.2%) | 153 (13.5%) | 0.38 |
| IL-6 Inhibitor Treatment | | | 1,081 (6.2%) | 793 (4.8%) | 288 (25.5%) | 0.6 |
| **Anthropometrics and Lab Values** | | | | | | |
| BMI | <18.5 | | 226 (1.3%) | 211 (1.3%) | 15 (1.3%) | 0.14 |
|  | 18.5 to 24.9 | | 5,346 (30.4%) | 5,054 (30.8%) | 292 (25.8%) |  |
|  | 25 to 29.9 | | 4,651 (26.5%) | 4,343 (26.4%) | 308 (27.2%) |  |
|  | ≥ 30 | | 4,752 (27.1%) | 4,391 (26.7%) | 361 (31.9%) |  |
|  | Missing | | 2,587 (14.7%) | 2,432 (14.8%) | 155 (13.7%) |  |
| Systolic Blood Pressure (mmHg) | < 140 | | 11,827 (67.3%) | 11,060 (67.3%) | 767 (67.8%) | 0.03 |
|  | ≥ 140 | | 5,702 (32.5%) | 5,339 (32.5%) | 363 (32.1%) |  |
|  | Missing | | 33 (0.2%) | 32 (0.2%) | 1 (0.1%) |  |
| Diastolic Blood Pressure (mmHg) | <90 | | 15,302 (87.1%) | 14,308 (87.1%) | 994 (87.9%) | 0.04 |
|  | ≥ 90 | | 2,227 (12.7%) | 2,091 (12.7%) | 136 (12.0%) |  |
|  | Missing | | 33 (0.2%) | 32 (0.2%) | 1 (0.1%) |  |
| SpO2 | | ≤ 94% | 6,473 (36.9%) | 5,809 (35.4%) | 664 (58.7%) | 0.49 |
|  |  | > 94% | 11,041 (62.9%) | 10,574 (64.4%) | 467 (41.3%) |  |
|  |  | Missing | 48 (0.3%) | 48 (0.3%) | 0 (0.0%) |  |
| Ferritin | | ≤ 800 ng/dl | 8,264 (47.1%) | 7,756 (47.2%) | 508 (44.9%) | 0.55 |
|  |  | > 800 ng/dl | 5,419 (30.9%) | 4,870 (29.6%) | 549 (48.5%) |  |
|  |  | Missing | 3,879 (22.1%) | 3,805 (23.2%) | 74 (6.5%) |  |
| C-reactive protein | | ≤ 30 mg/dl | 11,139 (63.4%) | 10,337 (62.9%) | 802 (70.9%) | 0.48 |
|  |  | > 30 mg/dl | 2,250 (12.8%) | 2,015 (12.3%) | 235 (20.8%) |  |
|  |  | Missing | 4,173 (23.8%) | 4,079 (24.8%) | 94 (8.3%) |  |
| D-Dimer | | ≤ 1000 ng/ml | 10,485 (59.7%) | 9,709 (59.1%) | 776 (68.6%) | 0.68 |
|  |  | > 1000 ng/ml | 1,463 (8.3%) | 1,218 (7.4%) | 245 (21.7%) |  |
|  |  | Missing | 5,614 (32.0%) | 5,504 (33.5%) | 110 (9.7%) |  |
| Creatinine | | < 1.5 mg/dL | 13,718 (78.1%) | 12,844 (78.2%) | 874 (77.3%) | 0.29 |
|  |  | ≥ 1.5 mg/dL | 3,204 (18.2%) | 2,948 (17.9%) | 256 (22.6%) |  |
|  |  | Missing | 640 (3.6%) | 639 (3.9%) | 1 (0.1%) |  |
| Lymphocyte count | | < 1.00 | 8,843 (50.4%) | 8,150 (49.6%) | 693 (61.3%) | 0.25 |
|  |  | ≥ 1.00 | 8,393 (47.8%) | 7,963 (48.5%) | 430 (38.0%) |  |
|  |  | Missing | 326 (1.9%) | 318 (1.9%) | 8 (0.7%) |  |
| Neutrophil count | | < 5.5 | 8,570 (48.8%) | 8,168 (49.7%) | 402 (35.5%) | 0.35 |
|  |  | ≥ 5.5 | 8,700 (49.5%) | 7,972 (48.5%) | 728 (64.4%) |  |
|  |  | Missing | 292 (1.7%) | 291 (1.8%) | 1 (0.1%) |  |
| Lactate Dehydrogenase (LDH) | | < 255 U/L | 2,532 (14.4%) | 2,451 (14.9%) | 81 (7.2%) | 0.8 |
|  |  | ≥ 255 U/L | 7,788 (44.3%) | 6,903 (42.0%) | 885 (78.2%) |  |
|  |  | Missing | 7,242 (41.2%) | 7,077 (43.1%) | 165 (14.6%) |  |
| Sodium | | < 136.0 | 8,568 (48.8%) | 7,864 (47.9%) | 704 (62.2%) | 0.38 |
|  |  | ≥ 136.0 | 8,369 (47.7%) | 7,942 (48.3%) | 427 (37.8%) |  |
|  |  | Missing | 625 (3.6%) | 625 (3.8%) | 0 (0.0%) |  |
| Potassium | | < 4.00 | 8,489 (48.3%) | 7,952 (48.4%) | 537 (47.5%) | 0.23 |
|  |  | ≥ 4.00 | 8,322 (47.4%) | 7,738 (47.1%) | 584 (51.6%) |  |
|  |  | Missing | 751 (4.3%) | 741 (4.5%) | 10 (0.9%) |  |
| Albumin | | < 2.5 mg/dL | 1,231 (7.0%) | 1,089 (6.6%) | 142 (12.6%) | 0.36 |
|  |  | ≥ 2.5 mg/dL | 15,603 (88.8%) | 14,614 (88.9%) | 989 (87.4%) |  |
|  |  | Missing | 728 (4.1%) | 728 (4.4%) | (0.0%) |  |
| White Blood Cell count | | < 6.16 | 6,013 (34.2%) | 5,731 (34.9%) | 282 (24.9%) | 0.23 |
|  |  | ≥ 6.16 | 11,498 (65.5%) | 10,649 (64.8%) | 849 (75.1%) |  |
|  |  | Missing | 51 (0.3%) | 51 (0.3%) | 0 (0.0%) |  |
| Platelet count | | < 150 | 14 (0.1%) | 14 (0.1%) | 0 (0.0%) | 0.06 |
|  |  | ≥ 150 | 17,472 (99.5%) | 16,343 (99.5%) | 1,129 (99.8%) |  |
|  |  | Missing | 76 (0.4%) | 74 (0.5%) | 2 (0.2%) |  |
| International Normalized Ratio (INR) | | ≤ 1.0 | 1,253 (7.1%) | 1,193 (7.3%) | 60 (5.3%) | 0.73 |
|  |  | >1.0 | 10,296 (58.6%) | 9,320 (56.7%) | 976 (86.3%) |  |
|  |  | Missing | 6,013 (34.2%) | 5,918 (36.0%) | 95 (8.4%) |  |
| Procalcitonin | | < 0.26 | 8,321 (47.4%) | 7,910 (48.1%) | 411 (36.3%) | 0.77 |
|  |  | ≥ 0.26 | 3,996 (22.8%) | 3,397 (20.7%) | 599 (53.0%) |  |
|  |  | Missing | 5,245 (29.9%) | 5,124 (31.2%) | 121 (10.7%) |  |
| Aspartate aminotransferase | | < 3x ULN | 15,678 (89.3%) | 14,694 (89.4%) | 984 (87.0%) | 0.34 |
|  |  | ≥ 3x ULN | 1,064 (6.1%) | 925 (5.6%) | 139 (12.3%) |  |
|  |  | Missing | 820 (4.7%) | 812 (4.9%) | 8 (0.7%) |  |
| Alanine aminotransferase | | < 3x ULN | 16,212 (92.3%) | 15,141 (92.1%) | 1,071 (94.7%) | 0.25 |
|  |  | ≥ 3x ULN | 448 (2.6%) | 402 (2.4%) | 46 (4.1%) |  |
|  |  | Missing | 902 (5.1%) | 888 (5.4%) | 14 (1.2%) |  |

Note: All values above reported as frequency and percentages unless otherwise noted.

Abbreviations: IQR – Interquartile Range; COPD – Chronic Obstructive Pulmonary Disease; CVD – Cardiovascular Disease; CKD – Chronic Kidney Disease; BMI – Body Mass Index; SpO2 – Oxygen Saturation

**Supplementary Table 3**: Absolute Standardized Differences Between Treated and Untreated Samples in the Propensity Score Matched Sample

|  | | | Total (n=2,262) | Did Not Receive Mechanical Ventilation (n=1,131) | Received Mechanical Ventilation (n=1,131) | Absolute Standardized Difference (ASD) |
| --- | --- | --- | --- | --- | --- | --- |
| Month of Admission | March 2020 through April 2020 | | 1,522 (67.3%) | 750 (66.3%) | 772 (68.3%) | 0.07 |
|  | May 2020 through July 2020 | | 159 (7.0%) | 89 (7.9%) | 70 (6.2%) |  |
|  | August 2020 through October 2020 | | 80 (3.5%) | 41 (3.6%) | 39 (3.4%) |  |
|  | November 2020 through January 2021 | | 501 (22.1%) | 251 (22.2%) | 250 (22.1%) |  |
| **Demographics** | | | | | | |
| Age; Median (IQR) | | | 63 (20) | 63 (21) | 63 (19) | 0.07 |
| Sex | | Female | 790 (34.9%) | 388 (34.3%) | 402 (35.5%) | 0.03 |
|  |  | Male | 1,472 (65.1%) | 743 (65.7%) | 729 (64.5%) |  |
| Race | | White | 883 (39.0%) | 434 (38.4%) | 449 (39.7%) | 0.03 |
|  |  | Black | 350 (15.5%) | 175 (15.5%) | 175 (15.5%) |  |
|  |  | Asian | 230 (10.2%) | 118 (10.4%) | 112 (9.9%) |  |
|  |  | Other/Multiracial | 671 (29.7%) | 338 (29.9%) | 333 (29.4%) |  |
|  |  | Unknown/Missing | 128 (5.7%) | 66 (5.8%) | 62 (5.5%) |  |
| Ethnicity | | Hispanic/Latinx | 503 (22.2%) | 249 (22.0%) | 254 (22.5%) | 0.04 |
|  |  | Non-Hispanic | 1,575 (69.6%) | 784 (69.3%) | 791 (69.9%) |  |
|  |  | Other/Unknown | 184 (8.1%) | 98 (8.7%) | 86 (7.6%) |  |
| Insurance | | Commercial | 822 (36.3%) | 402 (35.5%) | 420 (37.1%) | 0.07 |
|  |  | Medicare | 859 (38.0%) | 443 (39.2%) | 416 (36.8%) |  |
|  |  | Medicaid | 527 (23.3%) | 255 (22.5%) | 272 (24.0%) |  |
|  |  | Self-Pay | 11 (0.5%) | 6 (0.5%) | 5 (0.4%) |  |
|  |  | Other | 43 (1.9%) | 25 (2.2%) | 18 (1.6%) |  |
| **Comorbidity** | | | | | | |
| Smoking Status | | Current | 39 (1.7%) | 21 (1.9%) | 18 (1.6%) | 0.03 |
|  |  | Former | 240 (10.6%) | 124 (11.0%) | 116 (10.3%) |  |
|  |  | Never | 1,387 (61.3%) | 688 (60.8%) | 699 (61.8%) |  |
|  |  | Unknown | 547 (24.2%) | 273 (24.1%) | 274 (24.2%) |  |
|  |  | Missing | 49 (2.2%) | 25 (2.2%) | 24 (2.1%) |  |
| Asthma | | | 171 (7.6%) | 80 (7.1%) | 91 (8.0%) | 0.04 |
| COPD | | | 137 (6.1%) | 73 (6.5%) | 64 (5.7%) | 0.03 |
| Obstructive Sleep Apnea | | | 98 (4.3%) | 44 (3.9%) | 54 (4.8%) | 0.04 |
| Hypertension | | | 1,231 (54.4%) | 615 (54.4%) | 616 (54.5%) | 0.00 |
| Myocardial Infarction | | | 84 (3.7%) | 43 (3.8%) | 41 (3.6%) | 0.01 |
| Heart Failure | | | 237 (10.5%) | 123 (10.9%) | 114 (10.1%) | 0.03 |
| Stroke / Ischemic Disease | | | 47 (2.1%) | 25 (2.2%) | 22 (1.9%) | 0.02 |
| Aortic Aneurysm | | | 5 (0.2%) | 3 (0.3%) | 2 (0.2%) | 0.02 |
| CVD (all) | | | 290 (12.8%) | 147 (13.0%) | 143 (12.6%) | 0.01 |
| Diabetes Mellitus | | | 125 (5.5%) | 63 (5.6%) | 62 (5.5%) | 0.00 |
| CKD | | | 251 (11.1%) | 126 (11.1%) | 125 (11.1%) | 0.00 |
| Cancer | | | 178 (7.9%) | 95 (8.4%) | 83 (7.3%) | 0.04 |
| Dementia | | | 91 (4.0%) | 53 (4.7%) | 38 (3.4%) | 0.07 |
| Immunodeficiency | | | 16 (0.7%) | 8 (0.7%) | 8 (0.7%) | 0.00 |
| **Visit Details** | | | | | | |
| Length of Stay; Median (IQR) | | | 16 (23) | 13 (18) | 19 (27) | 0.37 |
| Antiviral Treatment | | | 1,819 (80.4%) | 896 (79.2%) | 923 (81.6%) | 0.06 |
| Anticoagulant Treatment | | | 2,242 (99.1%) | 1,121 (99.1%) | 1,121 (99.1%) | 0.00 |
| Corticosteroid Treatment | | | 1,763 (77.9%) | 872 (77.1%) | 891 (78.8%) | 0.04 |
| IL-1 Inhibitor Treatment | | | 314 (13.9%) | 161 (14.2%) | 153 (13.5%) | 0.02 |
| IL-6 Inhibitor Treatment | | | 553 (24.4%) | 265 (23.4%) | 288 (25.5%) | 0.05 |
| **Anthropometrics and Lab Values** | | | | | | |
| BMI | <18.5 | | 33 (1.5%) | 18 (1.6%) | 15 (1.3%) | 0.06 |
|  | 18.5 to 24.9 | | 563 (24.9%) | 271 (24.0%) | 292 (25.8%) |  |
|  | 25 to 29.9 | | 642 (28.4%) | 334 (29.5%) | 308 (27.2%) |  |
|  | ≥ 30 | | 712 (31.5%) | 351 (31.0%) | 361 (31.9%) |  |
|  | Missing | | 312 (13.8%) | 157 (13.9%) | 155 (13.7%) |  |
| Systolic Blood Pressure (mmHg) | < 140 | | 1,531 (67.7%) | 764 (67.6%) | 767 (67.8%) | 0.01 |
|  | ≥ 140 | | 729 (32.2%) | 366 (32.4%) | 363 (32.1%) |  |
|  | Missing | | 2 (0.1%) | 1 (0.1%) | 1 (0.1%) |  |
| Diastolic Blood Pressure (mmHg) | <90 | | 1,990 (88.0%) | 996 (88.1%) | 994 (87.9%) | 0.01 |
|  | ≥ 90 | | 270 (11.9%) | 134 (11.8%) | 136 (12.0%) |  |
|  | Missing | | 2 (0.1%) | 1 (0.1%) | 1 (0.1%) |  |
| SpO2 | | ≤ 94% | 1,309 (57.9%) | 645 (57.0%) | 664 (58.7%) | 0.03 |
|  |  | > 94% | 953 (42.1%) | 486 (43.0%) | 467 (41.3%) |  |
|  |  | Missing | 0 (0.0%) | 0 (0.0%) | 0 (0.0%) |  |
| Ferritin | | ≤ 800 ng/dl | 1,021 (45.1%) | 513 (45.4%) | 508 (44.9%) | 0.01 |
|  |  | > 800 ng/dl | 1,091 (48.2%) | 542 (47.9%) | 549 (48.5%) |  |
|  |  | Missing | 150 (6.6%) | 76 (6.7%) | 74 (6.5%) |  |
| C-reactive protein | | ≤ 30 mg/dl | 1,632 (72.1%) | 830 (73.4%) | 802 (70.9%) | 0.06 |
|  |  | > 30 mg/dl | 446 (19.7%) | 211 (18.7%) | 235 (20.8%) |  |
|  |  | Missing | 184 (8.1%) | 90 (8.0%) | 94 (8.3%) |  |
| D-Dimer | | ≤ 1000 ng/ml | 1,573 (69.5%) | 797 (70.5%) | 776 (68.6%) | 0.04 |
|  |  | > 1000 ng/ml | 476 (21.0%) | 231 (20.4%) | 245 (21.7%) |  |
|  |  | Missing | 213 (9.4%) | 103 (9.1%) | 110 (9.7%) |  |
| Creatinine | | < 1.5 mg/dL | 1,714 (75.8%) | 840 (74.3%) | 874 (77.3%) | 0.07 |
|  |  | ≥ 1.5 mg/dL | 546 (24.1%) | 290 (25.6%) | 256 (22.6%) |  |
|  |  | Missing | 2 (0.1%) | 1 (0.1%) | 1 (0.1%) |  |
| Lymphocyte count | | < 1.00 | 1,403 (62.0%) | 710 (62.8%) | 693 (61.3%) | 0.03 |
|  |  | ≥ 1.00 | 842 (37.2%) | 412 (36.4%) | 430 (38.0%) |  |
|  |  | Missing | 17 (0.8%) | 9 (0.8%) | 8 (0.7%) |  |
| Neutrophil count | | < 5.5 | 803 (35.5%) | 401 (35.5%) | 402 (35.5%) | 0.00 |
|  |  | ≥ 5.5 | 1,457 (64.4%) | 729 (64.5%) | 728 (64.4%) |  |
|  |  | Missing | 2 (0.1%) | 1 (0.1%) | 1 (0.1%) |  |
| Lactate Dehydrogenase (LDH) | | < 255 U/L | 169 (7.5%) | 88 (7.8%) | 81 (7.2%) | 0.05 |
|  |  | ≥ 255 U/L | 1,748 (77.3%) | 863 (76.3%) | 885 (78.2%) |  |
|  |  | Missing | 345 (15.3%) | 180 (15.9%) | 165 (14.6%) |  |
| Sodium | | < 136.0 | 1,372 (60.7%) | 668 (59.1%) | 704 (62.2%) | 0.07 |
|  |  | ≥ 136.0 | 890 (39.3%) | 463 (40.9%) | 427 (37.8%) |  |
|  |  | Missing | 0 (0.0%) | 0 (0.0%) | 0 (0.0%) |  |
| Potassium | | < 4.00 | 1,068 (47.2%) | 531 (46.9%) | 537 (47.5%) | 0.02 |
|  |  | ≥ 4.00 | 1,175 (51.9%) | 591 (52.3%) | 584 (51.6%) |  |
|  |  | Missing | 19 (0.8%) | 9 (0.8%) | 10 (0.9%) |  |
| Albumin | | < 2.5 mg/dL | 315 (13.9%) | 173 (15.3%) | 142 (12.6%) | 0.08 |
|  |  | ≥ 2.5 mg/dL | 1,947 (86.1%) | 958 (84.7%) | 989 (87.4%) |  |
|  |  | Missing | 0 (0.0%) | 0 (0.0%) | 0 (0.0%) |  |
| White Blood Cell count | | < 6.16 | 559 (24.7%) | 277 (24.5%) | 282 (24.9%) | 0.01 |
|  |  | ≥ 6.16 | 1,703 (75.3%) | 854 (75.5%) | 849 (75.1%) |  |
|  |  | Missing | 0 (0.0%) | 0 (0.0%) | 0 (0.0%) |  |
| Platelet count | | < 150 | 0 (0.0%) | 0 (0.0%) | 0 (0.0%) | 0.02 |
|  |  | ≥ 150 | 2,259 (99.9%) | 1,130 (99.9%) | 1,129 (99.8%) |  |
|  |  | Missing | 3 (0.1%) | 1 (0.1%) | 2 (0.2%) |  |
| International Normalized Ratio (INR) | | ≤ 1.0 | 112 (5.0%) | 52 (4.6%) | 60 (5.3%) | 0.04 |
|  |  | >1.0 | 1,952 (86.3%) | 976 (86.3%) | 976 (86.3%) |  |
|  |  | Missing | 198 (8.8%) | 103 (9.1%) | 95 (8.4%) |  |
| Procalcitonin | | < 0.26 | 776 (34.3%) | 365 (32.3%) | 411 (36.3%) | 0.09 |
|  |  | ≥ 0.26 | 1,230 (54.4%) | 631 (55.8%) | 599 (53.0%) |  |
|  |  | Missing | 256 (11.3%) | 135 (11.9%) | 121 (10.7%) |  |
| Aspartate aminotransferase | | < 3x ULN | 1,981 (87.6%) | 997 (88.2%) | 984 (87.0%) | 0.04 |
|  |  | ≥ 3x ULN | 267 (11.8%) | 128 (11.3%) | 139 (12.3%) |  |
|  |  | Missing | 14 (0.6%) | 6 (0.5%) | 8 (0.7%) |  |
| Alanine aminotransferase | | < 3x ULN | 2,150 (95.0%) | 1,079 (95.4%) | 1,071 (94.7%) | 0.03 |
|  |  | ≥ 3x ULN | 87 (3.8%) | 41 (3.6%) | 46 (4.1%) |  |
|  |  | Missing | 25 (1.1%) | 11 (1.0%) | 14 (1.2%) |  |

Note: All values above reported as frequency and percentages unless otherwise noted.

Abbreviations: IQR – Interquartile Range; COPD – Chronic Obstructive Pulmonary Disease; CVD – Cardiovascular Disease; CKD – Chronic Kidney Disease; BMI – Body Mass Index; SpO2 – Oxygen Saturation

**Supplementary Table 4:** Reasons for Readmission - Top Diagnoses by Type

| **Diagnosis Category** | **ICD-10 Code** | **Diagnosis Name** | **N in Category** |
| --- | --- | --- | --- |
| Abnormal Symptoms/Labs | R06.02 | Shortness of Breath | 109 |
|  | R09.02 | Hypoxemia | 98 |
|  | R41.82 | Altered mental status, unspecified | 33 |
|  | R53.1 | Weakness | 28 |
|  | R55 | Syncope and Collapse | 22 |
| COVID-19 | U07.1 | COVID-19 | 451 |
|  | B34.2 | Coronavirus infection, unspecified | 21 |
|  | U07.1,R53.81 | COVID-19 and Other Malaise | 6 |
|  | R53.81,U07.1 | Other Malaise and COVID-19 | 5 |
| Respiratory | J96.01 | Acute respiratory failure with hypoxia | 26 |
|  | J96.90 | Respiratory failure, unspecified | 18 |
|  | J96.91 | Respiratory failure, unspecified with hypoxia | 16 |
|  | J12.89 | Other viral pneumonia | 12 |
| Infectious Disease | A41.9 | Sepsis, unspecified | 54 |
|  | A41.89 | Other specified sepsis | 20 |
|  | B34.9 | Viral infection unspecified | 14 |
| Circulatory | I63.9 | Cerebral infarction, unspecified | 20 |
|  | I26.99 | Other pulmonary embolism without acute cor pulmonale | 18 |
|  | I95.9 | Hypotension, unspecified | 12 |
|  | I48.91 | Unspecified atrial fibrillation | 11 |

**Supplementary Table 5**: Absolute Standardized Differences Between MV and non-MV in the Readmitted Sample

|  | | | Total (n=1,994) | Did Not Receive Mechanical Ventilation (n=1,632) | Received Mechanical Ventilation (n=362) | Absolute Standardized Difference (ASD) |
| --- | --- | --- | --- | --- | --- | --- |
| Month of Admission | March 2020 through April 2020 | | 1,161 (58.2%) | 899 (55.1%) | 262 (72.4%) | 0.42 |
|  | May 2020 through July 2020 | | 166 (8.3%) | 154 (9.4%) | 12 (3.3%) |  |
|  | August 2020 through October 2020 | | 100 (5.0%) | 94 (5.8%) | 6 (1.7%) |  |
|  | November 2020 through January 2021 | | 567 (28.4%) | 485 (29.7%) | 82 (22.7%) |  |
| **Demographics** | | | | | | |
| Age; Median (IQR) | | | 68 (22) | 69 (22) | 62 (18) | 0.36 |
| Sex | | Female | 844 (42.3%) | 731 (44.8%) | 113 (31.2%) | 0.28 |
|  |  | Male | 1,150 (57.7%) | 901 (55.2%) | 249 (68.8%) |  |
| Race | | White | 928 (46.5%) | 803 (49.2%) | 125 (34.5%) | 0.39 |
|  |  | Black | 366 (18.4%) | 310 (19.0%) | 56 (15.5%) |  |
|  |  | Asian | 167 (8.4%) | 126 (7.7%) | 41 (11.3%) |  |
|  |  | Other/Multiracial | 453 (22.7%) | 338 (20.7%) | 115 (31.8%) |  |
|  |  | Unknown/Missing | 80 (4.0%) | 55 (3.4%) | 25 (6.9%) |  |
| Ethnicity | | Hispanic/Latinx | 344 (17.3%) | 257 (15.7%) | 87 (24.0%) | 0.3 |
|  |  | Non-Hispanic | 1,554 (77.9%) | 1,310 (80.3%) | 244 (67.4%) |  |
|  |  | Other/Unknown | 96 (4.8%) | 65 (4.0%) | 31 (8.6%) |  |
| Insurance | | Commercial | 554 (27.8%) | 412 (25.2%) | 142 (39.2%) | 0.43 |
|  |  | Medicare | 1,013 (50.8%) | 890 (54.5%) | 123 (34.0%) |  |
|  |  | Medicaid | 398 (20.0%) | 305 (18.7%) | 93 (25.7%) |  |
|  |  | Self-Pay | 6 (0.3%) | 5 (0.3%) | 1 (0.3%) |  |
|  |  | Other | 23 (1.2%) | 20 (1.2%) | 3 (0.8%) |  |
| **Comorbidity** | | | | | | |
| Smoking Status | | Current | 55 (2.8%) | 52 (3.2%) | 3 (0.8%) | 0.3 |
|  |  | Former | 270 (13.5%) | 237 (14.5%) | 33 (9.1%) |  |
|  |  | Never | 1,330 (66.7%) | 1,090 (66.8%) | 240 (66.3%) |  |
|  |  | Unknown | 306 (15.3%) | 228 (14.0%) | 78 (21.5%) |  |
|  |  | Missing | 33 (1.7%) | 25 (1.5%) | 8 (2.2%) |  |
| Asthma | | | 148 (7.4%) | 120 (7.4%) | 28 (7.7%) | 0.01 |
| COPD | | | 164 (8.2%) | 148 (9.1%) | 16 (4.4%) | 0.19 |
| Obstructive Sleep Apnea | | | 83 (4.2%) | 67 (4.1%) | 16 (4.4%) | 0.02 |
| Hypertension | | | 1,015 (50.9%) | 821 (50.3%) | 194 (53.6%) | 0.07 |
| Myocardial Infarction | | | 42 (2.1%) | 29 (1.8%) | 13 (3.6%) | 0.11 |
| Heart Failure | | | 228 (11.4%) | 201 (12.3%) | 27 (7.5%) | 0.16 |
| Stroke / Ischemic Disease | | | 43 (2.2%) | 41 (2.5%) | 2 (0.6%) | 0.16 |
| Aortic Aneurysm | | | 15 (0.8%) | 14 (0.9%) | 1 (0.3%) | 0.08 |
| CVD (all) | | | 258 (12.9%) | 221 (13.5%) | 37 (10.2%) | 0.1 |
| Diabetes Mellitus | | | 142 (7.1%) | 124 (7.6%) | 18 (5.0%) | 0.11 |
| CKD | | | 301 (15.1%) | 272 (16.7%) | 29 (8.0%) | 0.27 |
| Cancer | | | 198 (9.9%) | 176 (10.8%) | 22 (6.1%) | 0.17 |
| Dementia | | | 184 (9.2%) | 170 (10.4%) | 14 (3.9%) | 0.26 |
| Immunodeficiency | | | 14 (0.7%) | 12 (0.7%) | 2 (0.6%) | 0.02 |
| **Visit Details** | | | | | | |
| Length of Stay; Median (IQR) | | | 6 (9) | 5 (8) | 12 (21.75) | 0.81 |
| Antiviral Treatment | | | 1,162 (58.3%) | 844 (51.7%) | 318 (87.8%) | 0.86 |
| Anticoagulant Treatment | | | 1,858 (93.2%) | 1,500 (91.9%) | 358 (98.9%) | 0.34 |
| Corticosteroid Treatment | | | 1,061 (53.2%) | 775 (47.5%) | 286 (79.0%) | 0.69 |
| IL-1 Inhibitor Treatment | | | 109 (5.5%) | 46 (2.8%) | 63 (17.4%) | 0.5 |
| IL-6 Inhibitor Treatment | | | 160 (8.0%) | 68 (4.2%) | 92 (25.4%) | 0.63 |
| **Anthropometrics and Lab Values** | | | | | | |
| BMI | <18.5 | | 35 (1.8%) | 31 (1.9%) | 4 (1.1%) | 0.23 |
|  | 18.5 to 24.9 | | 636 (31.9%) | 535 (32.8%) | 101 (27.9%) |  |
|  | 25 to 29.9 | | 521 (26.1%) | 424 (26.0%) | 97 (26.8%) |  |
|  | ≥ 30 | | 475 (23.8%) | 363 (22.2%) | 112 (30.9%) |  |
|  | Missing | | 327 (16.4%) | 279 (17.1%) | 48 (13.3%) |  |
| Systolic Blood Pressure (mmHg) | < 140 | | 1,324 (66.4%) | 1,073 (65.7%) | 251 (69.3%) | 0.11 |
|  | ≥ 140 | | 664 (33.3%) | 553 (33.9%) | 111 (30.7%) |  |
|  | Missing | | 6 (0.3%) | 6 (0.4%) | 0 (0.0%) |  |
| Diastolic Blood Pressure (mmHg) | <90 | | 1,748 (87.7%) | 1,427 (87.4%) | 321 (88.7%) | 0.09 |
|  | ≥ 90 | | 240 (12.0%) | 199 (12.2%) | 41 (11.3%) |  |
|  | Missing | | 6 (0.3%) | 6 (0.4%) | 0 (0.0%) |  |
| SpO2 | | ≤ 94% | 744 (37.3%) | 513 (31.4%) | 231 (63.8%) | 0.69 |
|  |  | > 94% | 1,248 (62.6%) | 1,117 (68.4%) | 131 (36.2%) |  |
|  |  | Missing | 2 (0.1%) | 2 (0.1%) | 0 (0.0%) |  |
| Ferritin | | ≤ 800 ng/dl | 963 (48.3%) | 818 (50.1%) | 145 (40.1%) | 0.67 |
|  |  | > 800 ng/dl | 634 (31.8%) | 439 (26.9%) | 195 (53.9%) |  |
|  |  | Missing | 397 (19.9%) | 375 (23.0%) | 22 (6.1%) |  |
| C-reactive protein | | ≤ 30 mg/dl | 1,349 (67.7%) | 1,079 (66.1%) | 270 (74.6%) | 0.52 |
|  |  | > 30 mg/dl | 232 (11.6%) | 164 (10.0%) | 68 (18.8%) |  |
|  |  | Missing | 413 (20.7%) | 389 (23.8%) | 24 (6.6%) |  |
| D-Dimer | | ≤ 1000 ng/ml | 1,179 (59.1%) | 932 (57.1%) | 247 (68.2%) | 0.65 |
|  |  | > 1000 ng/ml | 230 (11.5%) | 151 (9.3%) | 79 (21.8%) |  |
|  |  | Missing | 585 (29.3%) | 549 (33.6%) | 36 (9.9%) |  |
| Creatinine | | < 1.5 mg/dL | 1,428 (71.6%) | 1,153 (70.6%) | 275 (76.0%) | 0.17 |
|  |  | ≥ 1.5 mg/dL | 553 (27.7%) | 466 (28.6%) | 87 (24.0%) |  |
|  |  | Missing | 13 (0.7%) | 13 (0.8%) | 0 (0.0%) |  |
| Lymphocyte count | | < 1.00 | 1,129 (56.6%) | 887 (54.4%) | 242 (66.9%) | 0.27 |
|  |  | ≥ 1.00 | 817 (41.0%) | 701 (43.0%) | 116 (32.0%) |  |
|  |  | Missing | 48 (2.4%) | 44 (2.7%) | 4 (1.1%) |  |
| Neutrophil count | | < 5.5 | 954 (47.8%) | 828 (50.7%) | 126 (34.8%) | 0.42 |
|  |  | ≥ 5.5 | 1,000 (50.2%) | 764 (46.8%) | 236 (65.2%) |  |
|  |  | Missing | 40 (2.0%) | 40 (2.5%) | 0 (0.0%) |  |
| Lactate Dehydrogenase (LDH) | | < 255 U/L | 291 (14.6%) | 271 (16.6%) | 20 (5.5%) | 0.96 |
|  |  | ≥ 255 U/L | 976 (48.9%) | 675 (41.4%) | 301 (83.1%) |  |
|  |  | Missing | 727 (36.5%) | 686 (42.0%) | 41 (11.3%) |  |
| Sodium | | < 136.0 | 976 (48.9%) | 737 (45.2%) | 239 (66.0%) | 0.44 |
|  |  | ≥ 136.0 | 1,004 (50.4%) | 881 (54.0%) | 123 (34.0%) |  |
|  |  | Missing | 14 (0.7%) | 14 (0.9%) | 0 (0.0%) |  |
| Potassium | | < 4.00 | 923 (46.3%) | 741 (45.4%) | 182 (50.3%) | 0.21 |
|  |  | ≥ 4.00 | 1,040 (52.2%) | 860 (52.7%) | 180 (49.7%) |  |
|  |  | Missing | 31 (1.6%) | 31 (1.9%) | 0 (0.0%) |  |
| Albumin | | < 2.5 mg/dL | 231 (11.6%) | 169 (10.4%) | 62 (17.1%) | 0.25 |
|  |  | ≥ 2.5 mg/dL | 1,744 (87.5%) | 1,444 (88.5%) | 300 (82.9%) |  |
|  |  | Missing | 19 (1.0%) | 19 (1.2%) | 0 (0.0%) |  |
| White Blood Cell count | | < 6.16 | 693 (34.8%) | 595 (36.5%) | 98 (27.1%) | 0.22 |
|  |  | ≥ 6.16 | 1,295 (64.9%) | 1,031 (63.2%) | 264 (72.9%) |  |
|  |  | Missing | 6 (0.3%) | 6 (0.4%) | 0 (0.0%) |  |
| Platelet count | | < 150 | 2 (0.1%) | 2 (0.1%) | 0 (0.0%) | 0.11 |
|  |  | ≥ 150 | 1,984 (99.5%) | 1,622 (99.4%) | 362 (100.0%) |  |
|  |  | Missing | 8 (0.4%) | 8 (0.5%) | 0 (0.0%) |  |
| International Normalized Ratio (INR) | | ≤ 1.0 | 128 (6.4%) | 111 (6.8%) | 17 (4.7%) | 0.57 |
|  |  | >1.0 | 1,318 (66.1%) | 1,009 (61.8%) | 309 (85.4%) |  |
|  |  | Missing | 548 (27.5%) | 512 (31.4%) | 36 (9.9%) |  |
| Procalcitonin | | < 0.26 | 859 (43.1%) | 735 (45.0%) | 124 (34.3%) | 0.69 |
|  |  | ≥ 0.26 | 606 (30.4%) | 408 (25.0%) | 198 (54.7%) |  |
|  |  | Missing | 529 (26.5%) | 489 (30.0%) | 40 (11.0%) |  |
| Aspartate aminotransferase | | < 3x ULN | 1,821 (91.3%) | 1,505 (92.2%) | 316 (87.3%) | 0.3 |
|  |  | ≥ 3x ULN | 142 (7.1%) | 96 (5.9%) | 46 (12.7%) |  |
|  |  | Missing | 31 (1.6%) | 31 (1.9%) | 0 (0.0%) |  |
| Alanine aminotransferase | | < 3x ULN | 1,912 (95.9%) | 1,560 (95.6%) | 352 (97.2%) | 0.2 |
|  |  | ≥ 3x ULN | 38 (1.9%) | 29 (1.8%) | 9 (2.5%) |  |
|  |  | Missing | 44 (2.2%) | 43 (2.6%) | 1 (0.3%) |  |

Note: All values above reported as frequency and percentages unless otherwise noted.

Abbreviations: IQR – Interquartile Range; COPD – Chronic Obstructive Pulmonary Disease; CVD – Cardiovascular Disease; CKD – Chronic Kidney Disease; BMI – Body Mass Index; SpO2 – Oxygen Saturation

**Supplementary Table 6**: Absolute Standardized Differences Between MV and non-MV Patients in the Readmitted Sample with 1-to-1 Propensity Score Matching

|  | | Total (n=724) | Did Not Receive Mechanical Ventilation (n=362) | Received Mechanical Ventilation (n=362) | Absolute Standardized Difference (ASD) |
| --- | --- | --- | --- | --- | --- |
| Month of Admission | March 2020 through April 2020 | 501 (69.2%) | 239 (66.0%) | 262 (72.4%) | 0.15 |
|  | May 2020 through July 2020 | 30 (4.1%) | 18 (5.0%) | 12 (3.3%) |  |
|  | August 2020 through October 2020 | 12 (1.7%) | 6 (1.7%) | 6 (1.7%) |  |
|  | November 2020 through January 2021 | 181 (25.0%) | 99 (27.3%) | 82 (22.7%) |  |
| **Demographics** | | | | | |
| Age; Median (IQR) | | 64 (18) | 65 (19) | 62 (18) | 0.18 |
| Sex | Female | 236 (32.6%) | 123 (34.0%) | 113 (31.2%) | 0.06 |
|  | Male | 488 (67.4%) | 239 (66.0%) | 249 (68.8%) |  |
| Race | White | 265 (36.6%) | 140 (38.7%) | 125 (34.5%) | 0.13 |
|  | Black | 121 (16.7%) | 65 (18.0%) | 56 (15.5%) |  |
|  | Asian | 76 (10.5%) | 35 (9.7%) | 41 (11.3%) |  |
|  | Other/Multiracial | 216 (29.8%) | 101 (27.9%) | 115 (31.8%) |  |
|  | Unknown/Missing | 46 (6.4%) | 21 (5.8%) | 25 (6.9%) |  |
| Ethnicity | Hispanic/Latinx | 167 (23.1%) | 80 (22.1%) | 87 (24.0%) | 0.09 |
|  | Non-Hispanic | 502 (69.3%) | 258 (71.3%) | 244 (67.4%) |  |
|  | Other/Unknown | 55 (7.6%) | 24 (6.6%) | 31 (8.6%) |  |
| Insurance | Commercial | 267 (36.9%) | 125 (34.5%) | 142 (39.2%) | 0.17 |
|  | Medicare | 273 (37.7%) | 150 (41.4%) | 123 (34.0%) |  |
|  | Medicaid | 174 (24.0%) | 81 (22.4%) | 93 (25.7%) |  |
|  | Self-Pay | 2 (0.3%) | 1 (0.3%) | 1 (0.3%) |  |
|  | Other | 8 (1.1%) | 5 (1.4%) | 3 (0.8%) |  |
| **Comorbidity** | | | | | |
| Smoking Status | Current | 8 (1.1%) | 5 (1.4%) | 3 (0.8%) | 0.11 |
|  | Former | 74 (10.2%) | 41 (11.3%) | 33 (9.1%) |  |
|  | Never | 480 (66.3%) | 240 (66.3%) | 240 (66.3%) |  |
|  | Unknown | 145 (20.0%) | 67 (18.5%) | 78 (21.5%) |  |
|  | Missing | 17 (2.3%) | 9 (2.5%) | 8 (2.2%) |  |
| Asthma | | 52 (7.2%) | 24 (6.6%) | 28 (7.7%) | 0.04 |
| COPD | | 31 (4.3%) | 15 (4.1%) | 16 (4.4%) | 0.01 |
| Obstructive Sleep Apnea | | 28 (3.9%) | 12 (3.3%) | 16 (4.4%) | 0.06 |
| Hypertension | | 380 (52.5%) | 186 (51.4%) | 194 (53.6%) | 0.04 |
| Myocardial Infarction | | 24 (3.3%) | 11 (3.0%) | 13 (3.6%) | 0.03 |
| Heart Failure | | 63 (8.7%) | 36 (9.9%) | 27 (7.5%) | 0.09 |
| Stroke / Ischemic Disease | | 5 (0.7%) | 3 (0.8%) | 2 (0.6%) | 0.03 |
| Aortic Aneurysm | | 3 (0.4%) | 2 (0.6%) | 1 (0.3%) | 0.04 |
| CVD (all) | | 78 (10.8%) | 41 (11.3%) | 37 (10.2%) | 0.04 |
| Diabetes Mellitus | | 43 (5.9%) | 25 (6.9%) | 18 (5.0%) | 0.08 |
| CKD | | 69 (9.5%) | 40 (11.0%) | 29 (8.0%) | 0.1 |
| Cancer | | 50 (6.9%) | 28 (7.7%) | 22 (6.1%) | 0.07 |
| Dementia | | 28 (3.9%) | 14 (3.9%) | 14 (3.9%) | 0.00 |
| Immunodeficiency | | 4 (0.6%) | 2 (0.6%) | 2 (0.6%) | 0.00 |
| **Visit Details** | | | | | |
| Length of Stay; Median (IQR) | | 10 (17) | 8 (12.75) | 12 (21.75) | 0.52 |
| Antiviral Treatment | | 625 (86.3%) | 307 (84.8%) | 318 (87.8%) | 0.09 |
| Anticoagulant Treatment | | 714 (98.6%) | 356 (98.3%) | 358 (98.9%) | 0.05 |
| Corticosteroid Treatment | | 550 (76.0%) | 264 (72.9%) | 286 (79.0%) | 0.14 |
| IL-1 Inhibitor Treatment | | 100 (13.8%) | 37 (10.2%) | 63 (17.4%) | 0.21 |
| IL-6 Inhibitor Treatment | | 144 (19.9%) | 52 (14.4%) | 92 (25.4%) | 0.28 |
| **Anthropometrics and Lab Values** | | | | | |
| BMI | <18.5 | 9 (1.2%) | 5 (1.4%) | 4 (1.1%) | 0.12 |
|  | 18.5 to 24.9 | 187 (25.8%) | 86 (23.8%) | 101 (27.9%) |  |
|  | 25 to 29.9 | 209 (28.9%) | 112 (30.9%) | 97 (26.8%) |  |
|  | ≥ 30 | 223 (30.8%) | 111 (30.7%) | 112 (30.9%) |  |
|  | Missing | 96 (13.3%) | 48 (13.3%) | 48 (13.3%) |  |
| Systolic Blood Pressure (mmHg) | < 140 | 498 (68.8%) | 247 (68.2%) | 251 (69.3%) | 0.02 |
|  | ≥ 140 | 226 (31.2%) | 115 (31.8%) | 111 (30.7%) |  |
|  | Missing | 0 (0.0%) | 0 (0.0%) | 0 (0.0%) |  |
| Diastolic Blood Pressure (mmHg) | <90 | 654 (90.3%) | 333 (92.0%) | 321 (88.7%) | 0.11 |
|  | ≥ 90 | 70 (9.7%) | 29 (8.0%) | 41 (11.3%) |  |
|  | Missing | 0 (0.0%) | 0 (0.0%) | 0 (0.0%) |  |
| SpO2 | ≤ 94% | 433 (59.8%) | 202 (55.8%) | 231 (63.8%) | 0.16 |
|  | > 94% | 291 (40.2%) | 160 (44.2%) | 131 (36.2%) |  |
|  | Missing | 0 (0.0%) | 0 (0.0%) | 0 (0.0%) |  |
| Ferritin | ≤ 800 ng/dl | 307 (42.4%) | 162 (44.8%) | 145 (40.1%) | 0.19 |
|  | > 800 ng/dl | 360 (49.7%) | 165 (45.6%) | 195 (53.9%) |  |
|  | Missing | 57 (7.9%) | 35 (9.7%) | 22 (6.1%) |  |
| C-reactive protein | ≤ 30 mg/dl | 545 (75.3%) | 275 (76.0%) | 270 (74.6%) | 0.06 |
|  | > 30 mg/dl | 128 (17.7%) | 60 (16.6%) | 68 (18.8%) |  |
|  | Missing | 51 (7.0%) | 27 (7.5%) | 24 (6.6%) |  |
| D-Dimer | ≤ 1000 ng/ml | 491 (67.8%) | 244 (67.4%) | 247 (68.2%) | 0.18 |
|  | > 1000 ng/ml | 142 (19.6%) | 63 (17.4%) | 79 (21.8%) |  |
|  | Missing | 91 (12.6%) | 55 (15.2%) | 36 (9.9%) |  |
| Creatinine | < 1.5 mg/dL | 533 (73.6%) | 258 (71.3%) | 275 (76.0%) | 0.11 |
|  | ≥ 1.5 mg/dL | 191 (26.4%) | 104 (28.7%) | 87 (24.0%) |  |
|  | Missing | 0 (0.0%) | 0 (0.0%) | 0 (0.0%) |  |
| Lymphocyte count | < 1.00 | 485 (67.0%) | 243 (67.1%) | 242 (66.9%) | 0.03 |
|  | ≥ 1.00 | 232 (32.0%) | 116 (32.0%) | 116 (32.0%) |  |
|  | Missing | 7 (1.0%) | 3 (0.8%) | 4 (1.1%) |  |
| Neutrophil count | < 5.5 | 276 (38.1%) | 150 (41.4%) | 126 (34.8%) | 0.14 |
|  | ≥ 5.5 | 448 (61.9%) | 212 (58.6%) | 236 (65.2%) |  |
|  | Missing | 0 (0.0%) | 0 (0.0%) | 0 (0.0%) |  |
| Lactate Dehydrogenase (LDH) | < 255 U/L | 49 (6.8%) | 29 (8.0%) | 20 (5.5%) | 0.19 |
|  | ≥ 255 U/L | 575 (79.4%) | 274 (75.7%) | 301 (83.1%) |  |
|  | Missing | 100 (13.8%) | 59 (16.3%) | 41 (11.3%) |  |
| Sodium | < 136.0 | 450 (62.2%) | 211 (58.3%) | 239 (66.0%) | 0.16 |
|  | ≥ 136.0 | 274 (37.8%) | 151 (41.7%) | 123 (34.0%) |  |
|  | Missing | 0 (0.0%) | 0 (0.0%) | 0 (0.0%) |  |
| Potassium | < 4.00 | 353 (48.8%) | 171 (47.2%) | 182 (50.3%) | 0.06 |
|  | ≥ 4.00 | 371 (51.2%) | 191 (52.8%) | 180 (49.7%) |  |
|  | Missing | 0 (0.0%) | 0 (0.0%) | 0 (0.0%) |  |
| Albumin | < 2.5 mg/dL | 120 (16.6%) | 58 (16.0%) | 62 (17.1%) | 0.03 |
|  | ≥ 2.5 mg/dL | 604 (83.4%) | 304 (84.0%) | 300 (82.9%) |  |
|  | Missing | 0 (0.0%) | 0 (0.0%) | 0 (0.0%) |  |
| White Blood Cell count | < 6.16 | 210 (29.0%) | 112 (30.9%) | 98 (27.1%) | 0.09 |
|  | ≥ 6.16 | 514 (71.0%) | 250 (69.1%) | 264 (72.9%) |  |
|  | Missing | 0 (0.0%) | 0 (0.0%) | 0 (0.0%) |  |
| Platelet count | < 150 | 0 (0.0%) | 0 (0.0%) | 0 (0.0%) | 0.00 |
|  | ≥ 150 | 724 (100.0%) | 362 (100.0%) | 362 (100.0%) |  |
|  | Missing | 0 (0.0%) | 0 (0.0%) | 0 (0.0%) |  |
| International Normalized Ratio (INR) | ≤ 1.0 | 37 (5.1%) | 20 (5.5%) | 17 (4.7%) | 0.22 |
|  | >1.0 | 589 (81.4%) | 280 (77.3%) | 309 (85.4%) |  |
|  | Missing | 98 (13.5%) | 62 (17.1%) | 36 (9.9%) |  |
| Procalcitonin | < 0.26 | 258 (35.6%) | 134 (37.0%) | 124 (34.3%) | 0.19 |
|  | ≥ 0.26 | 367 (50.7%) | 169 (46.7%) | 198 (54.7%) |  |
|  | Missing | 99 (13.7%) | 59 (16.3%) | 40 (11.0%) |  |
| Aspartate aminotransferase | < 3x ULN | 641 (88.5%) | 325 (89.8%) | 316 (87.3%) | 0.08 |
|  | ≥ 3x ULN | 83 (11.5%) | 37 (10.2%) | 46 (12.7%) |  |
|  | Missing | 0 (0.0%) | 0 (0.0%) | 0 (0.0%) |  |
| Alanine aminotransferase | < 3x ULN | 702 (97.0%) | 350 (96.7%) | 352 (97.2%) | 0.03 |
|  | ≥ 3x ULN | 20 (2.8%) | 11 (3.0%) | 9 (2.5%) |  |
|  | Missing | 2 (0.3%) | 1 (0.3%) | 1 (0.3%) |  |

Note: All values above reported as frequency and percentages unless otherwise noted.

Abbreviations: IQR – Interquartile Range; COPD – Chronic Obstructive Pulmonary Disease; CVD – Cardiovascular Disease; CKD – Chronic Kidney Disease; BMI – Body Mass Index; SpO2 – Oxygen Saturation

**Supplementary Table 7:** Reasons for Readmission in the Sample with 1-to-1 propensity matching

|  | | Total (n=724) | Did Not Receive Mechanical Ventilation (n=362) | Received Mechanical Ventilation (n=362) | Comparison p-value ‡ |
| --- | --- | --- | --- | --- | --- |
| Reason for Readmission | Abnormal Symptoms and Labs | 173 (23.9%) | 109 (30.1%) | 64 (17.7%) | <.001 |
|  | Birth | 2 (0.3%) | 0 (0.0%) | 2 (0.6%) | .479 |
|  | Blood Disease | 17 (2.3%) | 10 (2.8%) | 7 (1.9%) | .624 |
|  | Circulatory Issues | 44 (6.1%) | 33 (9.1%) | 11 (3.0%) | .001 |
|  | COVID-19 | 235 (32.5%) | 91 (25.1%) | 144 (39.8%) | <.001 |
|  | Digestive | 21 (2.9%) | 15 (4.1%) | 6 (1.7%) | .076 |
|  | Endocrine | 10 (1.4%) | 5 (1.4%) | 5 (1.4%) | >.999 |
|  | Eyes, Ears, and Skin | 4 (0.6%) | 4 (1.1%) | 0 (0.0%) | .133 |
|  | Genitourinary | 11 (1.5%) | 10 (2.8%) | 1 (0.3%) | .015 |
|  | Infectious Disease | 48 (6.6%) | 17 (4.7%) | 31 (8.6%) | .052 |
|  | Injury | 9 (1.2%) | 4 (1.1%) | 5 (1.4%) | >.999 |
|  | Mental | 6 (0.8%) | 5 (1.4%) | 1 (0.3%) | .219 |
|  | Muscular | 3 (0.4%) | 0 (0.0%) | 3 (0.8%) | .247 |
|  | Nervous System | 12 (1.7%) | 4 (1.1%) | 8 (2.2%) | .383 |
|  | Other | 16 (2.2%) | 7 (1.9%) | 9 (2.5%) | .800 |
|  | Pregnancy | 4 (0.6%) | 4 (1.1%) | 0 (0.0%) | .133 |
|  | Respiratory | 109 (15.1%) | 44 (12.2%) | 65 (18.0%) | .038 |

Note: All values above reported as frequency and percentages unless otherwise noted.

‡ Chi-squared tests used to generate p-values

**Supplementary Table 8a**: Absolute Standardized Differences Between MV and non-MV Patients Prior to Propensity Score Matching/Inverse Probability Weighting in the Black/African-American sample

|  | | | Total (n=3,231) | Did Not Receive Mechanical Ventilation (n=3,056) | Received Mechanical Ventilation (n=175) | Absolute Standardized Difference (ASD) |
| --- | --- | --- | --- | --- | --- | --- |
| Month of Admission | March 2020 through April 2020 | | 1,934 (59.9%) | 1,803 (59.0%) | 131 (74.9%) | 0.35 |
|  | May 2020 through July 2020 | | 318 (9.8%) | 308 (10.1%) | 10 (5.7%) |  |
|  | August 2020 through October 2020 | | 124 (3.8%) | 121 (4.0%) | 3 (1.7%) |  |
|  | November 2020 through January 2021 | | 855 (26.5%) | 824 (27.0%) | 31 (17.7%) |  |
| **Demographics** | | | | | | |
| Age; Median (IQR) | | | 62 (23) | 62 (23) | 62 (19.5) | 0.00 |
| Sex | | Female | 1,714 (53.0%) | 1,638 (53.6%) | 76 (43.4%) | 0.20 |
|  |  | Male | 1,517 (47.0%) | 1,418 (46.4%) | 99 (56.6%) |  |
| Ethnicity | | Hispanic/Latinx | 48 (1.5%) | 45 (1.5%) | 3 (1.7%) | 0.12 |
|  |  | Non-Hispanic | 3,155 (97.6%) | 2,987 (97.7%) | 168 (96.0%) |  |
|  |  | Other/Unknown | 28 (0.9%) | 24 (0.8%) | 4 (2.3%) |  |
| Insurance | | Commercial | 1,370 (42.4%) | 1,290 (42.2%) | 80 (45.7%) | 0.22 |
|  |  | Medicare | 1,234 (38.2%) | 1,179 (38.6%) | 55 (31.4%) |  |
|  |  | Medicaid | 545 (16.9%) | 507 (16.6%) | 38 (21.7%) |  |
|  |  | Self-Pay | 16 (0.5%) | 15 (0.5%) | 1 (0.6%) |  |
|  |  | Other | 66 (2.0%) | 65 (2.1%) | 1 (0.6%) |  |
| **Comorbidity** | | | | | | |
| Smoking Status | | Current | 99 (3.1%) | 97 (3.2%) | 2 (1.1%) | 0.6 |
|  |  | Former | 308 (9.5%) | 296 (9.7%) | 12 (6.9%) |  |
|  |  | Never | 2,461 (76.2%) | 2,358 (77.2%) | 103 (58.9%) |  |
|  |  | Unknown | 317 (9.8%) | 266 (8.7%) | 51 (29.1%) |  |
|  |  | Missing | 46 (1.4%) | 39 (1.3%) | 7 (4.0%) |  |
| Asthma | | | 304 (9.4%) | 285 (9.3%) | 19 (10.9%) | 0.05 |
| COPD | | | 169 (5.2%) | 160 (5.2%) | 9 (5.1%) | 0.00 |
| Obstructive Sleep Apnea | | | 106 (3.3%) | 97 (3.2%) | 9 (5.1%) | 0.1 |
| Hypertension | | | 1,933 (59.8%) | 1,818 (59.5%) | 115 (65.7%) | 0.13 |
| Myocardial Infarction | | | 50 (1.5%) | 41 (1.3%) | 9 (5.1%) | 0.22 |
| Heart Failure | | | 305 (9.4%) | 280 (9.2%) | 25 (14.3%) | 0.16 |
| Stroke / Ischemic Disease | | | 62 (1.9%) | 57 (1.9%) | 5 (2.9%) | 0.07 |
| Aortic Aneurysm | | | 9 (0.3%) | 9 (0.3%) | 0 (0.0%) | 0.08 |
| CVD (all) | | | 338 (10.5%) | 306 (10.0%) | 32 (18.3%) | 0.24 |
| Diabetes Mellitus | | | 189 (5.8%) | 177 (5.8%) | 12 (6.9%) | 0.04 |
| CKD | | | 506 (15.7%) | 478 (15.6%) | 28 (16.0%) | 0.01 |
| Cancer | | | 250 (7.7%) | 233 (7.6%) | 17 (9.7%) | 0.07 |
| Dementia | | | 178 (5.5%) | 169 (5.5%) | 9 (5.1%) | 0.02 |
| Immunodeficiency | | | 19 (0.6%) | 19 (0.6%) | 0 (0.0%) | 0.11 |
| **Visit Details** | | | | | | |
| Length of Stay; Median (IQR) | | | 5 (7) | 5 (6) | 17 (25) | 0.96 |
| Antiviral Treatment | | | 1,775 (54.9%) | 1,644 (53.8%) | 131 (74.9%) | 0.45 |
| Anticoagulant Treatment | | | 2,916 (90.3%) | 2,744 (89.8%) | 172 (98.3%) | 0.36 |
| Corticosteroid Treatment | | | 1,335 (41.3%) | 1,204 (39.4%) | 131 (74.9%) | 0.77 |
| IL-1 Inhibitor Treatment | | | 154 (4.8%) | 129 (4.2%) | 25 (14.3%) | 0.35 |
| IL-6 Inhibitor Treatment | | | 175 (5.4%) | 135 (4.4%) | 40 (22.9%) | 0.56 |
| **Anthropometrics and Lab Values** | | | | | | |
| BMI | <18.5 | | 48 (1.5%) | 44 (1.4%) | 4 (2.3%) | 0.09 |
|  | 18.5 to 24.9 | | 904 (28.0%) | 859 (28.1%) | 45 (25.7%) |  |
|  | 25 to 29.9 | | 752 (23.3%) | 712 (23.3%) | 40 (22.9%) |  |
|  | ≥ 30 | | 1,085 (33.6%) | 1,025 (33.5%) | 60 (34.3%) |  |
|  | Missing | | 442 (13.7%) | 416 (13.6%) | 26 (14.9%) |  |
| Systolic Blood Pressure (mmHg) | < 140 | | 2,046 (63.3%) | 1,932 (63.2%) | 114 (65.1%) | 0.07 |
|  | ≥ 140 | | 1,177 (36.4%) | 1,117 (36.6%) | 60 (34.3%) |  |
|  | Missing | | 8 (0.2%) | 7 (0.2%) | 1 (0.6%) |  |
| Diastolic Blood Pressure (mmHg) | <90 | | 2,669 (82.6%) | 2,520 (82.5%) | 149 (85.1%) | 0.1 |
|  | ≥ 90 | | 554 (17.1%) | 529 (17.3%) | 25 (14.3%) |  |
|  | Missing | | 8 (0.2%) | 7 (0.2%) | 1 (0.6%) |  |
| SpO2 | | ≤ 94% | 1,015 (31.4%) | 927 (30.3%) | 88 (50.3%) | 0.42 |
|  |  | > 94% | 2,215 (68.6%) | 2,128 (69.6%) | 87 (49.7%) |  |
|  |  | Missing | 1 (0.0%) | 1 (0.0%) | 0 (0.0%) |  |
| Ferritin | | ≤ 800 ng/dl | 1,494 (46.2%) | 1,406 (46.0%) | 88 (50.3%) | 0.52 |
|  |  | > 800 ng/dl | 969 (30.0%) | 894 (29.3%) | 75 (42.9%) |  |
|  |  | Missing | 768 (23.8%) | 756 (24.7%) | 12 (6.9%) |  |
| C-reactive protein | | ≤ 30 mg/dl | 1,805 (55.9%) | 1,692 (55.4%) | 113 (64.6%) | 0.42 |
|  |  | > 30 mg/dl | 589 (18.2%) | 546 (17.9%) | 43 (24.6%) |  |
|  |  | Missing | 837 (25.9%) | 818 (26.8%) | 19 (10.9%) |  |
| D-Dimer | | ≤ 1000 ng/ml | 1,720 (53.2%) | 1,615 (52.8%) | 105 (60.0%) | 0.71 |
|  |  | > 1000 ng/ml | 366 (11.3%) | 316 (10.3%) | 50 (28.6%) |  |
|  |  | Missing | 1,145 (35.4%) | 1,125 (36.8%) | 20 (11.4%) |  |
| Creatinine | | < 1.5 mg/dL | 2,195 (67.9%) | 2,088 (68.3%) | 107 (61.1%) | 0.29 |
|  |  | ≥ 1.5 mg/dL | 961 (29.7%) | 893 (29.2%) | 68 (38.9%) |  |
|  |  | Missing | 75 (2.3%) | 75 (2.5%) | 0 (0.0%) |  |
| Lymphocyte count | | < 1.00 | 1,380 (42.7%) | 1,292 (42.3%) | 88 (50.3%) | 0.25 |
|  |  | ≥ 1.00 | 1,785 (55.2%) | 1,698 (55.6%) | 87 (49.7%) |  |
|  |  | Missing | 66 (2.0%) | 66 (2.2%) | 0 (0.0%) |  |
| Neutrophil count | | < 5.5 | 1,776 (55.0%) | 1,708 (55.9%) | 68 (38.9%) | 0.43 |
|  |  | ≥ 5.5 | 1,390 (43.0%) | 1,283 (42.0%) | 107 (61.1%) |  |
|  |  | Missing | 65 (2.0%) | 65 (2.1%) | 0 (0.0%) |  |
| Lactate Dehydrogenase (LDH) | | < 255 U/L | 366 (11.3%) | 356 (11.6%) | 10 (5.7%) | 0.65 |
|  |  | ≥ 255 U/L | 1,583 (49.0%) | 1,448 (47.4%) | 135 (77.1%) |  |
|  |  | Missing | 1,282 (39.7%) | 1,252 (41.0%) | 30 (17.1%) |  |
| Sodium | | < 136.0 | 1,436 (44.4%) | 1,351 (44.2%) | 85 (48.6%) | 0.23 |
|  |  | ≥ 136.0 | 1,720 (53.2%) | 1,630 (53.3%) | 90 (51.4%) |  |
|  |  | Missing | 75 (2.3%) | 75 (2.5%) | 0 (0.0%) |  |
| Potassium | | < 4.00 | 1,454 (45.0%) | 1,375 (45.0%) | 79 (45.1%) | 0.16 |
|  |  | ≥ 4.00 | 1,669 (51.7%) | 1,575 (51.5%) | 94 (53.7%) |  |
|  |  | Missing | 108 (3.3%) | 106 (3.5%) | 2 (1.1%) |  |
| Albumin | | < 2.5 mg/dL | 278 (8.6%) | 247 (8.1%) | 31 (17.7%) | 0.38 |
|  |  | ≥ 2.5 mg/dL | 2,861 (88.5%) | 2,717 (88.9%) | 144 (82.3%) |  |
|  |  | Missing | 92 (2.8%) | 92 (3.0%) | 0 (0.0%) |  |
| White Blood Cell count | | < 6.16 | 1,211 (37.5%) | 1,165 (38.1%) | 46 (26.3%) | 0.26 |
|  |  | ≥ 6.16 | 2,014 (62.3%) | 1,885 (61.7%) | 129 (73.7%) |  |
|  |  | Missing | 6 (0.2%) | 6 (0.2%) | 0 (0.0%) |  |
| Platelet count | | < 150 | 1 (0.0%) | 1 (0.0%) | 0 (0.0%) | 0.09 |
|  |  | ≥ 150 | 3,219 (99.6%) | 3,044 (99.6%) | 175 (100.0%) |  |
|  |  | Missing | 11 (0.3%) | 11 (0.4%) | 0 (0.0%) |  |
| International Normalized Ratio (INR) | | ≤ 1.0 | 229 (7.1%) | 221 (7.2%) | 8 (4.6%) | 0.74 |
|  |  | >1.0 | 1,966 (60.8%) | 1,811 (59.3%) | 155 (88.6%) |  |
|  |  | Missing | 1,036 (32.1%) | 1,024 (33.5%) | 12 (6.9%) |  |
| Procalcitonin | | < 0.26 | 1,273 (39.4%) | 1,227 (40.2%) | 46 (26.3%) | 0.67 |
|  |  | ≥ 0.26 | 916 (28.4%) | 815 (26.7%) | 101 (57.7%) |  |
|  |  | Missing | 1,042 (32.3%) | 1,014 (33.2%) | 28 (16.0%) |  |
| Aspartate aminotransferase | | < 3x ULN | 2,885 (89.3%) | 2,734 (89.5%) | 151 (86.3%) | 0.22 |
|  |  | ≥ 3x ULN | 224 (6.9%) | 203 (6.6%) | 21 (12.0%) |  |
|  |  | Missing | 122 (3.8%) | 119 (3.9%) | 3 (1.7%) |  |
| Alanine aminotransferase | | < 3x ULN | 3,024 (93.6%) | 2,857 (93.5%) | 167 (95.4%) | 0.17 |
|  |  | ≥ 3x ULN | 66 (2.0%) | 61 (2.0%) | 5 (2.9%) |  |
|  |  | Missing | 141 (4.4%) | 138 (4.5%) | 3 (1.7%) |  |

Note: All values above reported as frequency and percentages unless otherwise noted.

Abbreviations: IQR – Interquartile Range; COPD – Chronic Obstructive Pulmonary Disease; CVD – Cardiovascular Disease; CKD – Chronic Kidney Disease; BMI – Body Mass Index; SpO2 – Oxygen Saturation

**Supplementary Table 9b**: Absolute Standardized Differences Between MV and non-MV Patients in the Black/African-American sample after 1-to-1 propensity score matching

|  | | | Total (n=350) | Did Not Receive Mechanical Ventilation (n=175) | Received Mechanical Ventilation (n=175) | Absolute Standardized Difference (ASD) |
| --- | --- | --- | --- | --- | --- | --- |
| Month of Admission | March 2020 through April 2020 | | 266 (76.0%) | 135 (77.1%) | 131 (74.9%) | 0.12 |
|  | May 2020 through July 2020 | | 18 (5.1%) | 8 (4.6%) | 10 (5.7%) |  |
|  | August 2020 through October 2020 | | 4 (1.1%) | 1 (0.6%) | 3 (1.7%) |  |
|  | November 2020 through January 2021 | | 62 (17.7%) | 31 (17.7%) | 31 (17.7%) |  |
| **Demographics** | | | | | | |
| Age; Median (IQR) | | | 63 (21) | 63 (22) | 62 (19.5) | 0.04 |
| Sex | | Female | 150 (42.9%) | 74 (42.3%) | 76 (43.4%) | 0.02 |
|  |  | Male | 200 (57.1%) | 101 (57.7%) | 99 (56.6%) |  |
| Ethnicity | | Hispanic/Latinx | 4 (1.1%) | 1 (0.6%) | 3 (1.7%) | 0.11 |
|  |  | Non-Hispanic | 337 (96.3%) | 169 (96.6%) | 168 (96.0%) |  |
|  |  | Other/Unknown | 9 (2.6%) | 5 (2.9%) | 4 (2.3%) |  |
| Insurance | | Commercial | 163 (46.6%) | 83 (47.4%) | 80 (45.7%) | 0.14 |
|  |  | Medicare | 105 (30.0%) | 50 (28.6%) | 55 (31.4%) |  |
|  |  | Medicaid | 75 (21.4%) | 37 (21.1%) | 38 (21.7%) |  |
|  |  | Self-Pay | 4 (1.1%) | 3 (1.7%) | 1 (0.6%) |  |
|  |  | Other | 3 (0.9%) | 2 (1.1%) | 1 (0.6%) |  |
| **Comorbidity** | | | | | | |
| Smoking Status | | Current | 5 (1.4%) | 3 (1.7%) | 2 (1.1%) | 0.16 |
|  |  | Former | 21 (6.0%) | 9 (5.1%) | 12 (6.9%) |  |
|  |  | Never | 216 (61.7%) | 113 (64.6%) | 103 (58.9%) |  |
|  |  | Unknown | 97 (27.7%) | 46 (26.3%) | 51 (29.1%) |  |
|  |  | Missing | 11 (3.1%) | 4 (2.3%) | 7 (4.0%) |  |
| Asthma | | | 39 (11.1%) | 20 (11.4%) | 19 (10.9%) | 0.02 |
| COPD | | | 17 (4.9%) | 8 (4.6%) | 9 (5.1%) | 0.03 |
| Obstructive Sleep Apnea | | | 15 (4.3%) | 6 (3.4%) | 9 (5.1%) | 0.08 |
| Hypertension | | | 222 (63.4%) | 107 (61.1%) | 115 (65.7%) | 0.10 |
| Myocardial Infarction | | | 16 (4.6%) | 7 (4.0%) | 9 (5.1%) | 0.05 |
| Heart Failure | | | 51 (14.6%) | 26 (14.9%) | 25 (14.3%) | 0.02 |
| Stroke / Ischemic Disease | | | 12 (3.4%) | 7 (4.0%) | 5 (2.9%) | 0.06 |
| Aortic Aneurysm | | | 0 (0.0%) | 0 (0.0%) | 0 (0.0%) | NA |
| CVD (all) | | | 61 (17.4%) | 29 (16.6%) | 32 (18.3%) | 0.05 |
| Diabetes Mellitus | | | 24 (6.9%) | 12 (6.9%) | 12 (6.9%) | 0.00 |
| CKD | | | 59 (16.9%) | 31 (17.7%) | 28 (16.0%) | 0.05 |
| Cancer | | | 32 (9.1%) | 15 (8.6%) | 17 (9.7%) | 0.04 |
| Dementia | | | 19 (5.4%) | 10 (5.7%) | 9 (5.1%) | 0.03 |
| Immunodeficiency | | | 0 (0.0%) | 0 (0.0%) | 0 (0.0%) | NA |
| **Visit Details** | | | | | | |
| Length of Stay; Median (IQR) | | | 14 (21) | 12 (16.5) | 17 (25) | 0.26 |
| Antiviral Treatment | | | 267 (76.3%) | 136 (77.7%) | 131 (74.9%) | 0.07 |
| Anticoagulant Treatment | | | 344 (98.3%) | 172 (98.3%) | 172 (98.3%) | 0.00 |
| Corticosteroid Treatment | | | 262 (74.9%) | 131 (74.9%) | 131 (74.9%) | 0.00 |
| IL-1 Inhibitor Treatment | | | 54 (15.4%) | 29 (16.6%) | 25 (14.3%) | 0.06 |
| IL-6 Inhibitor Treatment | | | 84 (24.0%) | 44 (25.1%) | 40 (22.9%) | 0.05 |
| **Anthropometrics and Lab Values** | | | | | | |
| BMI | <18.5 | | 7 (2.0%) | 3 (1.7%) | 4 (2.3%) | 0.09 |
|  | 18.5 to 24.9 | | 89 (25.4%) | 44 (25.1%) | 45 (25.7%) |  |
|  | 25 to 29.9 | | 84 (24.0%) | 44 (25.1%) | 40 (22.9%) |  |
|  | ≥ 30 | | 122 (34.9%) | 62 (35.4%) | 60 (34.3%) |  |
|  | Missing | | 48 (13.7%) | 22 (12.6%) | 26 (14.9%) |  |
| Systolic Blood Pressure (mmHg) | < 140 | | 228 (65.1%) | 114 (65.1%) | 114 (65.1%) | 0.11 |
|  | ≥ 140 | | 121 (34.6%) | 61 (34.9%) | 60 (34.3%) |  |
|  | Missing | | 1 (0.3%) | 0 (0.0%) | 1 (0.6%) |  |
| Diastolic Blood Pressure (mmHg) | <90 | | 298 (85.1%) | 149 (85.1%) | 149 (85.1%) | 0.11 |
|  | ≥ 90 | | 51 (14.6%) | 26 (14.9%) | 25 (14.3%) |  |
|  | Missing | | 1 (0.3%) | 0 (0.0%) | 1 (0.6%) |  |
| SpO2 | | ≤ 94% | 185 (52.9%) | 97 (55.4%) | 88 (50.3%) | 0.10 |
|  |  | > 94% | 165 (47.1%) | 78 (44.6%) | 87 (49.7%) |  |
|  |  | Missing | 0 (0.0%) | 0 (0.0%) | 0 (0.0%) |  |
| Ferritin | | ≤ 800 ng/dl | 168 (48.0%) | 80 (45.7%) | 88 (50.3%) | 0.15 |
|  |  | > 800 ng/dl | 162 (46.3%) | 87 (49.7%) | 75 (42.9%) |  |
|  |  | Missing | 20 (5.7%) | 8 (4.6%) | 12 (6.9%) |  |
| C-reactive protein | | ≤ 30 mg/dl | 231 (66.0%) | 118 (67.4%) | 113 (64.6%) | 0.10 |
|  |  | > 30 mg/dl | 86 (24.6%) | 43 (24.6%) | 43 (24.6%) |  |
|  |  | Missing | 33 (9.4%) | 14 (8.0%) | 19 (10.9%) |  |
| D-Dimer | | ≤ 1000 ng/ml | 218 (62.3%) | 113 (64.6%) | 105 (60.0%) | 0.13 |
|  |  | > 1000 ng/ml | 98 (28.0%) | 48 (27.4%) | 50 (28.6%) |  |
|  |  | Missing | 34 (9.7%) | 14 (8.0%) | 20 (11.4%) |  |
| Creatinine | | < 1.5 mg/dL | 206 (58.9%) | 99 (56.6%) | 107 (61.1%) | 0.09 |
|  |  | ≥ 1.5 mg/dL | 144 (41.1%) | 76 (43.4%) | 68 (38.9%) |  |
|  |  | Missing | 0 (0.0%) | 0 (0.0%) | 0 (0.0%) |  |
| Lymphocyte count | | < 1.00 | 187 (53.4%) | 99 (56.6%) | 88 (50.3%) | 0.13 |
|  |  | ≥ 1.00 | 163 (46.6%) | 76 (43.4%) | 87 (49.7%) |  |
|  |  | Missing | 0 (0.0%) | 0 (0.0%) | 0 (0.0%) |  |
| Neutrophil count | | < 5.5 | 135 (38.6%) | 67 (38.3%) | 68 (38.9%) | 0.01 |
|  |  | ≥ 5.5 | 215 (61.4%) | 108 (61.7%) | 107 (61.1%) |  |
|  |  | Missing | 0 (0.0%) | 0 (0.0%) | 0 (0.0%) |  |
| Lactate Dehydrogenase (LDH) | | < 255 U/L | 18 (5.1%) | 8 (4.6%) | 10 (5.7%) | 0.09 |
|  |  | ≥ 255 U/L | 276 (78.9%) | 141 (80.6%) | 135 (77.1%) |  |
|  |  | Missing | 56 (16.0%) | 26 (14.9%) | 30 (17.1%) |  |
| Sodium | | < 136.0 | 164 (46.9%) | 79 (45.1%) | 85 (48.6%) | 0.07 |
|  |  | ≥ 136.0 | 186 (53.1%) | 96 (54.9%) | 90 (51.4%) |  |
|  |  | Missing | 0 (0.0%) | 0 (0.0%) | 0 (0.0%) |  |
| Potassium | | < 4.00 | 153 (43.7%) | 74 (42.3%) | 79 (45.1%) | 0.07 |
|  |  | ≥ 4.00 | 192 (54.9%) | 98 (56.0%) | 94 (53.7%) |  |
|  |  | Missing | 5 (1.4%) | 3 (1.7%) | 2 (1.1%) |  |
| Albumin | | < 2.5 mg/dL | 64 (18.3%) | 33 (18.9%) | 31 (17.7%) | 0.03 |
|  |  | ≥ 2.5 mg/dL | 286 (81.7%) | 142 (81.1%) | 144 (82.3%) |  |
|  |  | Missing | 0 (0.0%) | 0 (0.0%) | 0 (0.0%) |  |
| White Blood Cell count | | < 6.16 | 94 (26.9%) | 48 (27.4%) | 46 (26.3%) | 0.03 |
|  |  | ≥ 6.16 | 256 (73.1%) | 127 (72.6%) | 129 (73.7%) |  |
|  |  | Missing | 0 (0.0%) | 0 (0.0%) | 0 (0.0%) |  |
| Platelet count | | < 150 | 0 (0.0%) | 0 (0.0%) | 0 (0.0%) | 0.00 |
|  |  | ≥ 150 | 350 (100.0%) | 175 (100.0%) | 175 (100.0%) |  |
|  |  | Missing | 0 (0.0%) | 0 (0.0%) | 0 (0.0%) |  |
| International Normalized Ratio (INR) | | ≤ 1.0 | 13 (3.7%) | 5 (2.9%) | 8 (4.6%) | 0.09 |
|  |  | >1.0 | 314 (89.7%) | 159 (90.9%) | 155 (88.6%) |  |
|  |  | Missing | 23 (6.6%) | 11 (6.3%) | 12 (6.9%) |  |
| Procalcitonin | | < 0.26 | 86 (24.6%) | 40 (22.9%) | 46 (26.3%) | 0.08 |
|  |  | ≥ 0.26 | 205 (58.6%) | 104 (59.4%) | 101 (57.7%) |  |
|  |  | Missing | 59 (16.9%) | 31 (17.7%) | 28 (16.0%) |  |
| Aspartate aminotransferase | | < 3x ULN | 298 (85.1%) | 147 (84.0%) | 151 (86.3%) | 0.07 |
|  |  | ≥ 3x ULN | 46 (13.1%) | 25 (14.3%) | 21 (12.0%) |  |
|  |  | Missing | 6 (1.7%) | 3 (1.7%) | 3 (1.7%) |  |
| Alanine aminotransferase | | < 3x ULN | 331 (94.6%) | 164 (93.7%) | 167 (95.4%) | 0.09 |
|  |  | ≥ 3x ULN | 13 (3.7%) | 8 (4.6%) | 5 (2.9%) |  |
|  |  | Missing | 6 (1.7%) | 3 (1.7%) | 3 (1.7%) |  |

Note: All values above reported as frequency and percentages unless otherwise noted.

Abbreviations: IQR – Interquartile Range; COPD – Chronic Obstructive Pulmonary Disease; CVD – Cardiovascular Disease; CKD – Chronic Kidney Disease; BMI – Body Mass Index; SpO2 – Oxygen Saturation

**Supplemental Table 8c:** Cox Proportional Hazards Regression for Primary (Inpatient Readmission) and Secondary Outcomes (All-cause Mortality) for the Sample Identifying as Black/African-American

| **Primary Outcome**  *Readmission* | **Category** | **Frequencies** | | **Cox Proportional Hazards Regression** | |
| --- | --- | --- | --- | --- | --- |
|  | **Unadjusted Sample** | | | | |
|  | Non-MV | Total  (N=3,231) | Readmitted  (n=366; 11.3%) | Hazard Ratio (95% CI) | Hazard Ratio ‡ (95% CI) |
|  |  | 3,056 (94.6%) | 310 (10.1%) | REF | REF |
|  | MV | 175 (5.4%) | 56 (32.0%) | 3.54***  (2.66 to 4.71) | 3.80***  (2.70 to 5.34) |
|  | **Propensity Score Matched Sample** | | | | |
|  | Non-MV | Total  (n=350) | Readmitted  (n=78; 22.3%) | Hazard Ratio (95% CI) | Hazard Ratio ‡ (95% CI) |
|  |  | 175 (50%) | 22 (12.6%) | REF | REF |
|  | MV | 175 (50%) | 56 (32.0%) | 2.92***  (1.78 to 4.78) | 3.25***  (1.96 to 5.40) |
| **Secondary Outcome**  *All-cause Mortality* | **Unadjusted Sample** | | | | |
|  | Non-MV | Total  (n=3,231) | Mortality  (n=175; 5.4%) | Odds Ratio (95% CI) | Odds Ratio ‡ (95% CI) |
|  |  | 3,056 (94.6%) | 97 (3.2%) | REF | REF |
|  | MV | 175 (5.4%) | 29 (16.6%) | 5.62***  (3.71 to 8.52) | 9.89***  (5.99 to 16.33) |
|  | **Propensity Score Matched Sample** | | | | |
|  | Non-MV | Total  (n=350) | Mortality  (n=33; 9.4%) | Odds Ratio (95% CI) | Odds Ratio ‡ (95% CI) |
|  |  | 175 (50%) | 4 (2.3%) | REF | REF |
|  | MV | 175 (50%) | 29 (16.6%) | 7.75***  (2.72 to 22.04) | 9.92***  (3.39 to 28.99) |

*p<.05; **p<.01; ***p<.001

Note: MV- Mechanical Ventilation;

‡Covariates adjusted for include length of stay, month of admission, smoking status, insurance status, ethnicity, hypertension status, systolic blood pressure, diastolic blood pressure, SP02 category, ferritin, c-reactive protein, and D-Dimer. These covariate-adjusted models did not converge due to small sample sizes but are presented for completeness. Parameter estimates for covariate-adjusted models should be interpreted cautiously.

**Supplementary Table 9a**: Absolute Standardized Differences Between MV and non-MV Patients Prior to Propensity Score Matching/Inverse Probability Weighting in the Sample Treated with Corticosteroids

|  | | | Total (n=8,651) | Did Not Receive Mechanical Ventilation (n=7,760) | Received Mechanical Ventilation (n=891) | Absolute Standardized Difference (ASD) |
| --- | --- | --- | --- | --- | --- | --- |
| Month of Admission | March 2020 through April 2020 | | 3,284 (38.0%) | 2,685 (34.6%) | 599 (67.2%) | 0.70 |
|  | May 2020 through July 2020 | | 433 (5.0%) | 394 (5.1%) | 39 (4.4%) |  |
|  | August 2020 through October 2020 | | 473 (5.5%) | 444 (5.7%) | 29 (3.3%) |  |
|  | November 2020 through January 2021 | | 4,461 (51.6%) | 4,237 (54.6%) | 224 (25.1%) |  |
| **Demographics** | | | | | | |
| Age; Median (IQR) | | | 64 (22) | 64 (23) | 63 (19) | 0.10 |
| Sex | | Female | 3,815 (44.1%) | 3,496 (45.1%) | 319 (35.8%) | 0.19 |
|  |  | Male | 4,836 (55.9%) | 4,264 (54.9%) | 572 (64.2%) |  |
| Race | | White | 4,097 (47.4%) | 3,733 (48.1%) | 364 (40.9%) | 0.17 |
|  |  | Black | 1,335 (15.4%) | 1,204 (15.5%) | 131 (14.7%) |  |
|  |  | Asian | 733 (8.5%) | 646 (8.3%) | 87 (9.8%) |  |
|  |  | Other/Multiracial | 2,110 (24.4%) | 1,848 (23.8%) | 262 (29.4%) |  |
|  |  | Unknown/Missing | 376 (4.3%) | 329 (23.8%) | 47 (29.4%) |  |
| Ethnicity | | Hispanic/Latinx | 1,553 (18.0%) | 1,353 (17.4%) | 200 (22.4%) | 0.16 |
|  |  | Non-Hispanic | 6,599 (76.3%) | 5,974 (77.0%) | 625 (70.1%) |  |
|  |  | Other/Unknown | 499 (5.8%) | 433 (5.6%) | 66 (7.4%) |  |
| Insurance | | Commercial | 3,162 (36.6%) | 2,819 (36.3%) | 343 (38.5%) | 0.14 |
|  |  | Medicare | 3,664 (42.4%) | 3,334 (43.0%) | 330 (37.0%) |  |
|  |  | Medicaid | 1,649 (19.1%) | 1,449 (18.7%) | 200 (22.4%) |  |
|  |  | Self-Pay | 44 (0.5%) | 42 (0.5%) | 2 (0.2%) |  |
|  |  | Other | 132 (1.5%) | 116 (1.5%) | 16 (1.8%) |  |
| **Comorbidity** | | | | | | |
| Smoking Status | | Current | 185 (2.1%) | 171 (2.2%) | 14 (1.6%) | 0.44 |
|  |  | Former | 1,188 (13.7%) | 1,092 (14.1%) | 96 (10.8%) |  |
|  |  | Never | 6,339 (73.3%) | 5,782 (74.5%) | 557 (62.5%) |  |
|  |  | Unknown | 840 (9.7%) | 634 (8.2%) | 206 (23.1%) |  |
|  |  | Missing | 99 (1.1%) | 81 (1.0%) | 18 (2.0%) |  |
| Asthma | | | 890 (10.3%) | 811 (10.5%) | 79 (8.9%) | 0.05 |
| COPD | | | 828 (9.6%) | 765 (9.9%) | 63 (7.1%) | 0.10 |
| Obstructive Sleep Apnea | | | 382 (4.4%) | 335 (4.3%) | 47 (5.3%) | 0.04 |
| Hypertension | | | 4,514 (52.2%) | 4,014 (51.7%) | 500 (56.1%) | 0.09 |
| Myocardial Infarction | | | 112 (1.3%) | 89 (1.1%) | 23 (2.6%) | 0.11 |
| Heart Failure | | | 756 (8.7%) | 672 (8.7%) | 84 (9.4%) | 0.03 |
| Stroke / Ischemic Disease | | | 104 (1.2%) | 91 (1.2%) | 13 (1.5%) | 0.03 |
| Aortic Aneurysm | | | 39 (0.5%) | 37 (0.5%) | 2 (0.2%) | 0.04 |
| CVD (all) | | | 826 (9.5%) | 727 (9.4%) | 99 (11.1%) | 0.06 |
| Diabetes Mellitus | | | 412 (4.8%) | 367 (4.7%) | 45 (5.1%) | 0.01 |
| CKD | | | 867 (10.0%) | 774 (10.0%) | 93 (10.4%) | 0.02 |
| Cancer | | | 772 (8.9%) | 700 (9.0%) | 72 (8.1%) | 0.03 |
| Dementia | | | 463 (5.4%) | 433 (5.6%) | 30 (3.4%) | 0.11 |
| Immunodeficiency | | | 71 (0.8%) | 64 (0.8%) | 7 (0.8%) | 0.00 |
| **Visit Details** | | | | | | |
| Length of Stay; Median (IQR) | | | 7 (9) | 6 (7) | 21 (29.5) | 1.04 |
| Antiviral Treatment | | | 6,369 (73.6%) | 5,592 (72.1%) | 777 (87.2%) | 0.38 |
| Anticoagulant Treatment | | | 8,357 (96.6%) | 7,468 (96.2%) | 889 (99.8%) | 0.26 |
| IL-1 Inhibitor Treatment | | | 604 (7.0%) | 453 (5.8%) | 151 (16.9%) | 0.36 |
| IL-6 Inhibitor Treatment | | | 887 (10.3%) | 617 (8.0%) | 270 (30.3%) | 0.59 |
| **Anthropometrics and Lab Values** | | | | | | |
| BMI | <18.5 | | 83 (1.0%) | 74 (1.0%) | 9 (1.0%) | 0.12 |
|  | 18.5 to 24.9 | | 2,755 (31.8%) | 2,512 (32.4%) | 243 (27.3%) |  |
|  | 25 to 29.9 | | 2,289 (26.5%) | 2,048 (26.4%) | 241 (27.0%) |  |
|  | ≥ 30 | | 2,462 (28.5%) | 2,179 (28.1%) | 283 (31.8%) |  |
|  | Missing | | 1,062 (12.3%) | 947 (12.2%) | 115 (12.9%) |  |
| Systolic Blood Pressure (mmHg) | < 140 | | 5,853 (67.7%) | 5,258 (67.8%) | 595 (66.8%) | 0.05 |
|  | ≥ 140 | | 2,792 (32.3%) | 2,496 (32.2%) | 296 (33.2%) |  |
|  | Missing | | 6 (0.1%) | 6 (0.1%) | 0 (0.0%) |  |
| Diastolic Blood Pressure (mmHg) | <90 | | 7,616 (88.0%) | 6,825 (88.0%) | 791 (88.8%) | 0.05 |
|  | ≥ 90 | | 1,029 (11.9%) | 929 (12.0%) | 100 (11.2%) |  |
|  | Missing | | 6 (0.1%) | 6 (0.1%) | 0 (0.0%) |  |
| SpO2 | | ≤ 94% | 4,155 (48.0%) | 3,586 (46.2%) | 569 (63.9%) | 0.36 |
|  |  | > 94% | 4,481 (51.8%) | 4,159 (53.6%) | 322 (36.1%) |  |
|  |  | Missing | 15 (0.2%) | 15 (0.2%) | 0 (0.0%) |  |
| Ferritin | | ≤ 800 ng/dl | 4,404 (50.9%) | 4,003 (51.6%) | 401 (45.0%) | 0.39 |
|  |  | > 800 ng/dl | 3,406 (39.4%) | 2,941 (37.9%) | 465 (52.2%) |  |
|  |  | Missing | 841 (9.7%) | 816 (10.5%) | 25 (2.8%) |  |
| C-reactive protein | | ≤ 30 mg/dl | 6,336 (73.2%) | 5,685 (73.3%) | 651 (73.1%) | 0.27 |
|  |  | > 30 mg/dl | 1,324 (15.3%) | 1,134 (14.6%) | 190 (21.3%) |  |
|  |  | Missing | 991 (11.5%) | 941 (12.1%) | 50 (5.6%) |  |
| D-Dimer | | ≤ 1000 ng/ml | 6,316 (73.0%) | 5,671 (73.1%) | 645 (72.4%) | 0.51 |
|  |  | > 1000 ng/ml | 860 (9.9%) | 666 (8.6%) | 194 (21.8%) |  |
|  |  | Missing | 1,475 (17.1%) | 1,423 (18.3%) | 52 (5.8%) |  |
| Creatinine | | < 1.5 mg/dL | 6,995 (80.9%) | 6,300 (81.2%) | 695 (78.0%) | 0.21 |
|  |  | ≥ 1.5 mg/dL | 1,531 (17.7%) | 1,335 (17.2%) | 196 (22.0%) |  |
|  |  | Missing | 125 (1.4%) | 125 (1.6%) | 0 (0.0%) |  |
| Lymphocyte count | | < 1.00 | 5,072 (58.6%) | 4,506 (58.1%) | 566 (63.5%) | 0.12 |
|  |  | ≥ 1.00 | 3,475 (40.2%) | 3,157 (40.7%) | 318 (35.7%) |  |
|  |  | Missing | 104 (1.2%) | 97 (1.3%) | 7 (0.8%) |  |
| Neutrophil count | | < 5.5 | 4,121 (47.6%) | 3,811 (49.1%) | 310 (34.8%) | 0.33 |
|  |  | ≥ 5.5 | 4,452 (51.5%) | 3,872 (49.9%) | 580 (65.1%) |  |
|  |  | Missing | 78 (0.9%) | 77 (1.0%) | 1 (0.1%) |  |
| Lactate Dehydrogenase (LDH) | | < 255 U/L | 1,092 (12.6%) | 1,039 (13.4%) | 53 (5.9%) | 0.73 |
|  |  | ≥ 255 U/L | 4,703 (54.4%) | 3,963 (51.1%) | 740 (83.1%) |  |
|  |  | Missing | 2,856 (33.0%) | 2,758 (35.5%) | 98 (11.0%) |  |
| Sodium | | < 136.0 | 4,596 (53.1%) | 4,019 (51.8%) | 577 (64.8%) | 0.3 |
|  |  | ≥ 136.0 | 3,941 (45.6%) | 3,627 (46.7%) | 314 (35.2%) |  |
|  |  | Missing | 114 (1.3%) | 114 (1.5%) | 0 (0.0%) |  |
| Potassium | | < 4.00 | 4,318 (49.9%) | 3,893 (50.2%) | 425 (47.7%) | 0.11 |
|  |  | ≥ 4.00 | 4,147 (47.9%) | 3,691 (47.6%) | 456 (51.2%) |  |
|  |  | Missing | 186 (2.2%) | 176 (2.3%) | 10 (1.1%) |  |
| Albumin | | < 2.5 mg/dL | 653 (7.5%) | 544 (7.0%) | 109 (12.2%) | 0.26 |
|  |  | ≥ 2.5 mg/dL | 7,863 (90.9%) | 7,081 (91.3%) | 782 (87.8%) |  |
|  |  | Missing | 135 (1.6%) | 135 (1.7%) | 0 (0.0%) |  |
| White Blood Cell count | | < 6.16 | 3,026 (35.0%) | 2,799 (36.1%) | 227 (25.5%) | 0.23 |
|  |  | ≥ 6.16 | 5,619 (65.0%) | 4,955 (63.9%) | 664 (74.5%) |  |
|  |  | Missing | 6 (0.1%) | 6 (0.1%) | 0 (0.0%) |  |
| Platelet count | | < 150 | 13 (0.2%) | 13 (0.2%) | 0 (0.0%) | 0.06 |
|  |  | ≥ 150 | 8,615 (99.6%) | 7,726 (99.6%) | 889 (99.8%) |  |
|  |  | Missing | 23 (0.3%) | 21 (0.3%) | 2 (0.2%) |  |
| International Normalized Ratio (INR) | | ≤ 1.0 | 489 (5.7%) | 447 (5.8%) | 42 (4.7%) | 0.69 |
|  |  | >1.0 | 5,436 (62.8%) | 4,660 (60.1%) | 776 (87.1%) |  |
|  |  | Missing | 2,726 (31.5%) | 2,653 (34.2%) | 73 (8.2%) |  |
| Procalcitonin | | < 0.26 | 4,798 (55.5%) | 4,448 (57.3%) | 350 (39.3%) | 0.69 |
|  |  | ≥ 0.26 | 2,431 (28.1%) | 1,939 (25.0%) | 492 (55.2%) |  |
|  |  | Missing | 1,422 (16.4%) | 1,373 (17.7%) | 49 (5.5%) |  |
| Aspartate aminotransferase | | < 3x ULN | 7,877 (91.1%) | 7,101 (91.5%) | 776 (87.1%) | 0.23 |
|  |  | ≥ 3x ULN | 586 (6.8%) | 479 (6.2%) | 107 (12.0%) |  |
|  |  | Missing | 188 (2.2%) | 180 (2.3%) | 8 (0.9%) |  |
| Alanine aminotransferase | | < 3x ULN | 8,214 (94.9%) | 7,368 (94.9%) | 846 (94.9%) | 0.12 |
|  |  | ≥ 3x ULN | 226 (2.6%) | 192 (2.5%) | 34 (3.8%) |  |
|  |  | Missing | 201 (2.3%) | 200 (2.6%) | 1 (1.2%) |  |

Note: All values above reported as frequency and percentages unless otherwise noted.

Abbreviations: IQR – Interquartile Range; COPD – Chronic Obstructive Pulmonary Disease; CVD – Cardiovascular Disease; CKD – Chronic Kidney Disease; BMI – Body Mass Index; SpO2 – Oxygen Saturation

**Supplementary Table 9b**: Absolute Standardized Differences Between MV and non-MV Patients in the Sample Treated with Corticosteroids after 1-to-1 propensity score matching

|  | | | Total (n=1,782) | Did Not Receive Mechanical Ventilation (n=891) | Received Mechanical Ventilation (n=891) | Absolute Standardized Difference (ASD) |  |
| --- | --- | --- | --- | --- | --- | --- | --- |
| Month of Admission | March 2020 through April 2020 | | 1,198 (67.2%) | 599 (67.2%) | 599 (67.2%) | 0.02 |  |
|  | May 2020 through July 2020 | | 78 (4.4%) | 39 (4.4%) | 39 (4.4%) |  |  |
|  | August 2020 through October 2020 | | 55 (3.1%) | 26 (2.9%) | 29 (3.3%) |  |  |
|  | November 2020 through January 2021 | | 451 (25.3%) | 227 (25.5%) | 224 (25.1%) |  |  |
| **Demographics** | | | | | | | |
| Age; Median (IQR) | | | 63 (20) | 63 (20 | 63 (19) | 0.04 |  |
| Sex | | Female | 648 (36.4%) | 329 (36.9%) | 319 (35.8%) | 0.02 |  |
|  |  | Male | 1,134 (63.6%) | 562 (63.1%) | 572 (64.2%) |  |  |
| Race | | White | 699 (39.2%) | 335 (37.6%) | 364 (40.9%) | 0.13 |  |
|  |  | Black | 299 (16.8%) | 168 (18.9%) | 131 (14.7%) |  |  |
|  |  | Asian | 156 (8.8%) | 69 (7.7%) | 87 (9.8%) |  |  |
|  |  | Other/Multiracial | 534 (30.0%) | 272 (30.5%) | 262 (29.4%) |  |  |
|  |  | Unknown/Missing | 94 (5.3%) | 47 (5.3%) | 47 (5.3%) |  |  |
| Ethnicity | | Hispanic/Latinx | 410 (23.0%) | 210 (23.6%) | 200 (22.4%) | 0.03 |  |
|  |  | Non-Hispanic | 1,237 (69.4%) | 612 (68.7%) | 625 (70.1%) |  |  |
|  |  | Other/Unknown | 135 (7.6%) | 69 (7.7%) | 66 (7.4%) |  |  |
| Insurance | | Commercial | 683 (38.3%) | 340 (38.2%) | 343 (38.5%) | 0.04 |  |
|  |  | Medicare | 671 (37.7%) | 341 (38.3%) | 330 (37.0%) |  |  |
|  |  | Medicaid | 390 (21.9%) | 190 (21.3%) | 200 (22.4%) |  |  |
|  |  | Self-Pay | 4 (0.2%) | 2 (0.2%) | 2 (0.2%) |  |  |
|  |  | Other | 34 (1.9%) | 18 (2.0%) | 16 (1.8%) |  |  |
| **Comorbidity** | | | | | | | |
| Smoking Status | | Current | 34 (1.9%) | 20 (2.2%) | 14 (1.6%) | 0.09 |  |
|  |  | Former | 197 (11.1%) | 101 (11.3%) | 96 (10.8%) |  |  |
|  |  | Never | 1,117 (62.7%) | 560 (62.9%) | 557 (62.5%) |  |  |
|  |  | Unknown | 390 (21.9%) | 184 (20.7%) | 206 (23.1%) |  |  |
|  |  | Missing | 44 (2.5%) | 26 (2.9%) | 18 (2.0%) |  |  |
| Asthma | | | 161 (9.0%) | 82 (9.2%) | 79 (8.9%) | 0.01 |  |
| COPD | | | 124 (7.0%) | 61 (6.8%) | 63 (7.1%) | 0.01 |  |
| Obstructive Sleep Apnea | | | 89 (5.0%) | 42 (4.7%) | 47 (5.3%) | 0.03 |  |
| Hypertension | | | 975 (54.7%) | 475 (53.3%) | 500 (56.1%) | 0.06 |  |
| Myocardial Infarction | | | 44 (2.5%) | 21 (2.4%) | 23 (2.6%) | 0.01 |  |
| Heart Failure | | | 168 (9.4%) | 84 (9.4%) | 84 (9.4%) | 0.00 |  |
| Stroke / Ischemic Disease | | | 24 (1.3%) | 11 (1.2%) | 13 (1.5%) | 0.02 |  |
| Aortic Aneurysm | | | 6 (0.3%) | 4 (0.4%) | 2 (0.2%) | 0.04 |  |
| CVD (all) | | | 193 (10.8%) | 94 (10.5%) | 99 (11.1%) | 0.02 |  |
| Diabetes Mellitus | | | 86 (4.8%) | 41 (4.6%) | 45 (5.1%) | 0.02 |  |
| CKD | | | 204 (11.4%) | 111 (12.5%) | 93 (10.4%) | 0.06 |  |
| Cancer | | | 141 (7.9%) | 69 (7.7%) | 72 (8.1%) | 0.01 |  |
| Dementia | | | 64 (3.6%) | 34 (3.8%) | 30 (3.4%) | 0.02 |  |
| Immunodeficiency | | | 11 (0.6%) | 4 (0.4%) | 7 (0.8%) | 0.04 |  |
| **Visit Details** | | | | | | | |
| Length of Stay; Median (IQR) | | | 17 (24) | 14 (17) | 21 (29.5) | 0.47 |  |
| Antiviral Treatment | | | 1,550 (87.0%) | 773 (86.8%) | 777 (87.2%) | 0.01 |  |
| Anticoagulant Treatment | | | 1,777 (99.7%) | 888 (99.7%) | 889 (99.8%) | 0.02 |  |
| IL-1 Inhibitor Treatment | | | 305 (17.1%) | 154 (17.3%) | 151 (16.9%) | 0.01 |  |
| IL-6 Inhibitor Treatment | | | 510 (28.6%) | 240 (26.9%) | 270 (30.3%) | 0.07 |  |
| **Anthropometrics and Lab Values** | | | | | | | |
| BMI | <18.5 | | 20 (1.1%) | 11 (1.2%) | 9 (1.0%) | 0.03 |  |
|  | 18.5 to 24.9 | | 478 (26.8%) | 235 (26.4%) | 243 (27.3%) |  |  |
|  | 25 to 29.9 | | 488 (27.4%) | 247 (27.7%) | 241 (27.0%) |  |  |
|  | ≥ 30 | | 563 (31.6%) | 280 (31.4%) | 283 (31.8%) |  |  |
|  | Missing | | 233 (13.1%) | 118 (13.2%) | 115 (12.9%) |  |  |
| Systolic Blood Pressure (mmHg) | < 140 | | 1,200 (67.3%) | 605 (67.9%) | 595 (66.8%) | 0.02 |  |
|  | ≥ 140 | | 582 (32.7%) | 286 (32.1%) | 296 (33.2%) |  |  |
|  | Missing | | 0 (0.0%) | 0 (0.0%) | 0 (0.0%) |  |  |
| Diastolic Blood Pressure (mmHg) | <90 | | 1,583 (88.8%) | 792 (88.9%) | 791 (88.8%) | 0.00 |  |
|  | ≥ 90 | | 199 (11.2%) | 99 (11.1%) | 100 (11.2%) |  |  |
|  | Missing | | 0 (0.0%) | 0 (0.0%) | 0 (0.0%) |  |  |
| SpO2 | | ≤ 94% | 1,123 (63.0%) | 554 (62.2%) | 569 (63.9%) | 0.03 |  |
|  |  | > 94% | 659 (37.0%) | 337 (37.8%) | 322 (36.1%) |  |  |
|  |  | Missing | 0 (0.0%) | 0 (0.0%) | 0 (0.0%) |  |  |
| Ferritin | | ≤ 800 ng/dl | 809 (45.4%) | 408 (45.8%) | 401 (45.0%) | 0.02 |  |
|  |  | > 800 ng/dl | 923 (51.8%) | 458 (51.4%) | 465 (52.2%) |  |  |
|  |  | Missing | 50 (2.8%) | 25 (2.8%) | 25 (2.8%) |  |  |
| C-reactive protein | | ≤ 30 mg/dl | 1,326 (74.4%) | 675 (75.8%) | 651 (73.1%) | 0.06 |  |
|  |  | > 30 mg/dl | 358 (20.1%) | 168 (18.9%) | 190 (21.3%) |  |  |
|  |  | Missing | 98 (5.5%) | 48 (5.4%) | 50 (5.6%) |  |  |
| D-Dimer | | ≤ 1000 ng/ml | 1,307 (73.3%) | 662 (74.3%) | 645 (72.4%) | 0.05 |  |
|  |  | > 1000 ng/ml | 369 (20.7%) | 175 (19.6%) | 194 (21.8%) |  |  |
|  |  | Missing | 106 (5.9%) | 54 (6.1%) | 52 (5.8%) |  |  |
| Creatinine | | < 1.5 mg/dL | 1,354 (76.0%) | 659 (74.0%) | 695 (78.0%) | 0.09 |  |
|  |  | ≥ 1.5 mg/dL | 428 (24.0%) | 232 (26.0%) | 196 (22.0%) |  |  |
|  |  | Missing | 0 (0.0%) | 0 (0.0%) | 0 (0.0%) |  |  |
| Lymphocyte count | | < 1.00 | 1,153 (64.7%) | 587 (65.9%) | 566 (63.5%) | 0.06 |  |
|  |  | ≥ 1.00 | 612 (34.3%) | 294 (33.0%) | 318 (35.7%) |  |  |
|  |  | Missing | 17 (1.0%) | 10 (1.1%) | 7 (0.8%) |  |  |
| Neutrophil count | | < 5.5 | 616 (34.6%) | 306 (34.3%) | 310 (34.8%) | 0.01 |  |
|  |  | ≥ 5.5 | 1,164 (65.3%) | 584 (65.5%) | 580 (65.1%) |  |  |
|  |  | Missing | 2 (0.1%) | 1 (0.1%) | 1 (0.1%) |  |  |
| Lactate Dehydrogenase (LDH) | | < 255 U/L | 110 (6.2%) | 57 (6.4%) | 53 (5.9%) | 0.03 |  |
|  |  | ≥ 255 U/L | 1,482 (83.2%) | 742 (83.3%) | 740 (83.1%) |  |  |
|  |  | Missing | 190 (10.7%) | 92 (10.3%) | 98 (11.0%) |  |  |
| Sodium | | < 136.0 | 1,152 (64.6%) | 575 (64.5%) | 577 (64.8%) | 0.00 |  |
|  |  | ≥ 136.0 | 630 (35.4%) | 316 (35.5%) | 314 (35.2%) |  |  |
|  |  | Missing | 0 (0.0%) | 0 (0.0%) | 0 (0.0%) |  |  |
| Potassium | | < 4.00 | 838 (47.0%) | 413 (46.4%) | 425 (47.7%) | 0.03 |  |
|  |  | ≥ 4.00 | 925 (51.9%) | 469 (52.6%) | 456 (51.2%) |  |  |
|  |  | Missing | 19 (1.1%) | 9 (1.0%) | 10 (1.1%) |  |  |
| Albumin | | < 2.5 mg/dL | 236 (13.2%) | 127 (14.3%) | 109 (12.2%) | 0.06 |  |
|  |  | ≥ 2.5 mg/dL | 1,546 (86.8%) | 764 (85.7%) | 782 (87.8%) |  |  |
|  |  | Missing | 0 (0.0%) | 0 (0.0%) | 0 (0.0%) |  |  |
| White Blood Cell count | | < 6.16 | 455 (25.5%) | 228 (25.6%) | 227 (25.5%) | 0.00 |  |
|  |  | ≥ 6.16 | 1,327 (74.5%) | 663 (74.4%) | 664 (74.5%) |  |  |
|  |  | Missing | 0 (0.0%) | 0 (0.0%) | 0 (0.0%) |  |  |
| Platelet count | | < 150 | 0 (0.0%) | 0 (0.0%) | 0 (0.0%) | 0.00 |  |
|  |  | ≥ 150 | 1,778 (99.8%) | 889 (99.8%) | 889 (99.8%) |  |  |
|  |  | Missing | 4 (0.2%) | 2 (0.2%) | 2 (0.2%) |  |  |
| International Normalized Ratio (INR) | | ≤ 1.0 | 81 (4.5%) | 39 (4.4%) | 42 (4.7%) | 0.04 |  |
|  |  | >1.0 | 1,545 (86.7%) | 769 (86.3%) | 776 (87.1%) |  |  |
|  |  | Missing | 156 (8.8%) | 83 (9.3%) | 73 (8.2%) |  |  |
| Procalcitonin | | < 0.26 | 663 (37.2%) | 313 (35.1%) | 350 (39.3%) | 0.09 |  |
|  |  | ≥ 0.26 | 1,014 (56.9%) | 522 (58.6%) | 492 (55.2%) |  |  |
|  |  | Missing | 105 (5.9%) | 56 (6.3%) | 49 (5.5%) |  |  |
| Aspartate aminotransferase | | < 3x ULN | 1,561 (87.6%) | 785 (88.1%) | 776 (87.1%) | 0.03 |  |
|  |  | ≥ 3x ULN | 205 (11.5%) | 98 (11.0%) | 107 (12.0%) |  |  |
|  |  | Missing | 16 (0.9%) | 8 (0.9%) | 8 (0.9%) |  |  |
| Alanine aminotransferase | | < 3x ULN | 1,692 (94.9%) | 846 (94.9%) | 846 (94.9%) | 0.01 |  |
|  |  | ≥ 3x ULN | 69 (3.9%) | 35 (3.9%) | 34 (3.8%) |  |  |
|  |  | Missing | 21 (1.2%) | 10 (1.1%) | 11 (1.2%) |  |  |

Note: All values above reported as frequency and percentages unless otherwise noted.

Abbreviations: IQR – Interquartile Range; COPD – Chronic Obstructive Pulmonary Disease; CVD – Cardiovascular Disease; CKD – Chronic Kidney Disease; BMI – Body Mass Index; SpO2 – Oxygen Saturation

**Supplemental Table 9c:** Cox Proportional Hazards Regression for Primary (Inpatient Readmission) and Secondary Outcomes (All-cause Mortality) for the Sample Prescribed Corticosteroids during initial admission

| **Primary Outcome**  *Readmission* | **Category** | **Frequencies** | | **Cox Proportional Hazards Regression** | |
| --- | --- | --- | --- | --- | --- |
|  | **Unadjusted Sample** | | | | |
|  | Non-MV | Total  (N=8,651) | Readmitted  (n=891; 10.3%) | Hazard Ratio (95% CI) | Hazard Ratio ‡ (95% CI) |
|  |  | 7,760 (89.7%) | 775 (10.0%) | REF | REF |
|  | MV | 891 (10.3%) | 286 (32.1%) | 3.57***  (3.11 to 4.08) | 4.48***  (3.83 to 5.24) |
|  | **Propensity Score Matched Sample** | | | | |
|  | Non-MV | Total  (n=1,782) | Readmitted  (n=382; 21.4%) | Hazard Ratio (95% CI) | Hazard Ratio ‡ (95% CI) |
|  |  | 891 (50.0%) | 96 (10.8%) | REF | REF |
|  | MV | 891 (50.0%) | 286 (32.1%) | 3.37***  (2.68 to 4.25) | 3.83***  3.03 to 4.85) |
| **Secondary Outcome**  *All-cause Mortality* | **Unadjusted Sample** | | | | |
|  | Non-MV | Total  (n=8,651) | Mortality  (n=419; 4.8%) | Hazard Ratio (95% CI) | Hazard Ratio ‡ (95% CI) |
|  |  | 7,760 (89.7%) | 276 (3.6%) | REF | REF |
|  | MV | 891 (10.3%) | 143 (16.0%) | 4.69***  (3.83 to 5.74) | 6.39***  (5.06 to 8.07) |
|  | **Propensity Score Matched Sample** | | | | |
|  | Non-MV | Total  (n=1,782) | Mortality  (n=180; 10.1%) | Hazard Ratio (95% CI) | Hazard Ratio ‡ (95% CI) |
|  |  | 891 (50.0%) | 37 (4.2%) | REF | REF |
|  | MV | 891 (50.0%) | 143 (16.0%) | 4.13***  (2.88 to 5.93) | 5.30***  (3.68 to 7.63) |

*p<.05; **p<.01; ***p<.001; ‡ Covariate adjusted for length of stay

Note: MV- Mechanical Ventilation;

‡ Covariates adjusted for include length of stay and race
